# Diverse, evolving conformer populations drive distinct phenotypes in frontotemporal lobar degeneration caused by the same *MAPT*-P301L mutation

**DOI:** 10.1007/s00401-020-02148-4

**Published:** 2020-03-26

**Authors:** Nathalie Daude, Chae Kim, Sang-Gyun Kang, Ghazaleh Eskandari-Sedighi, Tracy Haldiman, Jing Yang, Shelaine C. Fleck, Erik Gomez-Cardona, Zhuang Zhuang Han, Sergi Borrego-Ecija, Serene Wohlgemuth, Olivier Julien, Holger Wille, Laura Molina-Porcel, Ellen Gelpi, Jiri G. Safar, David Westaway

**Affiliations:** 1 Centre for Prions and Protein Folding Diseases, University of Alberta, 204 Brain and Aging Research Building, Edmonton, T6G 2M8 Canada; 2 Department of Pathology, Case Western Reserve University, Institute of Pathology Building, Rm 406, 2085 Adelbert Road, Cleveland, OH 44106-4907 USA; 3 Department of Neurology, Case Western Reserve University, Institute of Pathology Building, Rm 406, 2085 Adelbert Road, Cleveland, OH 44106-4907 USA; 4 Neurological Tissue Bank of the Biobanc, Hospital Clinic, IDIBAPS, Barcelona, Spain; 5 Neurology Department, Hospital Clinic, IDIBAPS, Barcelona, Spain; 6 Division of Neuropathology and Neurochemistry, Department of Neurology, Medical University of Vienna, Vienna, Austria; 7 Department of Biochemistry, University of Alberta, Edmonton, AB Canada

**Keywords:** Tauopathy, Aging/P301L mutation, Conformation, Focal pathology, Seeding, Strain ensembles, Transgenic mouse

## Abstract

Tau protein accumulation is a common denominator of major dementias, but this process is inhomogeneous, even when triggered by the same germline mutation. We considered stochastic misfolding of human tau conformers followed by templated conversion of native monomers as an underlying mechanism and derived sensitive conformational assays to test this concept. Assessments of brains from aged TgTau^P301L^ transgenic mice revealed a prodromal state and three distinct signatures for misfolded tau. Frontotemporal lobar degeneration (FTLD)-MAPT-P301L patients with different clinical phenotypes also displayed three signatures, two resembling those found in TgTau^P301L^ mice. As physicochemical and cell bioassays confirmed diverse tau strains in the mouse and human brain series, we conclude that evolution of diverse tau conformers is intrinsic to the pathogenesis of this uni-allelic form of tauopathy. In turn, effective therapeutic interventions in FTLD will need to address evolving repertoires of misfolded tau species rather than singular, static molecular targets.

**Electronic supplementary material:**

The online version of this article (10.1007/s00401-020-02148-4) contains supplementary material, which is available to authorized users.

## Introduction

**Fig. 1:**
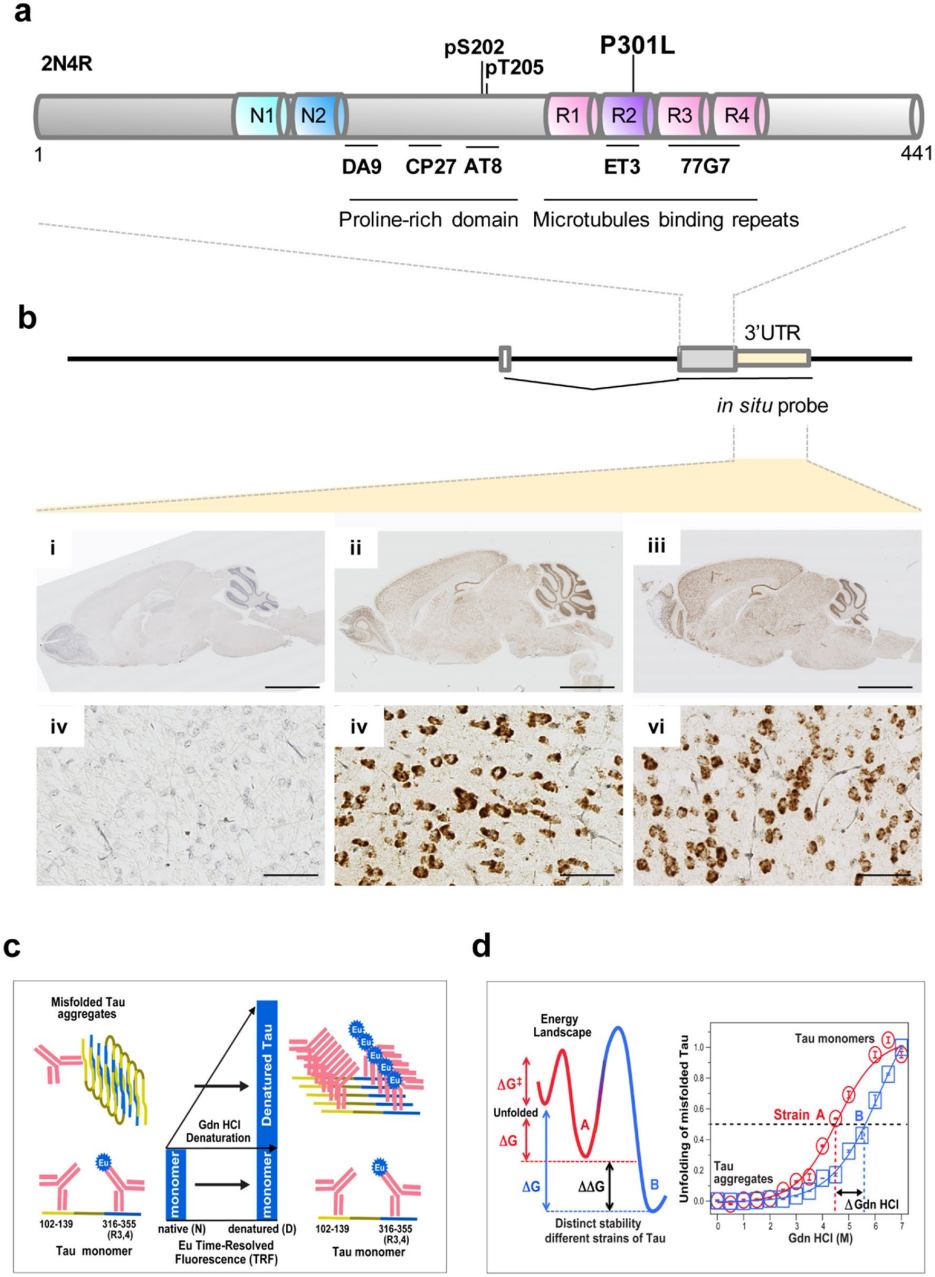
Tau protein structure, transgene expression, and tools for monitoring different conformers. **a** Features within the tau protein, post translational modifications and antibody epitopes; not to scale and with microtubule-binding repeats exaggerated. **b** Expression of 2N4R tau transcripts in the mouse brain. Upper: transgene structure with spliced mRNA and position of the hamster PrP 3′UTR detected by the ISH probe. Lower: ISH of adult mouse brains performed with the 3′UTR hybridization probe. (i–iii), low-power views showing non-Tg, young Tg and aged Tg mice, respectively. Brown DAB staining indicates broad neuroanatomical expression of the transgene-encoded mRNA versus non-Tg control. Scale bar = 2.5 mm. (iv–vi) Higher power views of sections in (i–iii) indicating mRNA accumulation in neuronal cell bodies. Scale bar = 50 μm. **c** Capture and detection antibodies for detecting aggregates of misfolded tau and unstructured tau monomers after complete denaturation in sandwich formatted CDI [85]. *Eu* europium label. **d** Energy landscape and schematic representation of two prion-like strains of misfolded tau denoted A and B in CSA using sequential denaturation in Gdn HCl [23, 52, 85]

After Alzheimer’s Disease (AD), Frontotemporal Dementia (FTD) is the most common dementia seen in subjects over 65 [54]. FTD encompasses different clinical presentations, most commonly presenting as a behavioral variant FTD (bvFTD), as well as other sub-varieties such as primary progressive aphasia (PPA) (itself with three sub-varieties [66, 71]) and may be associated with atypical parkinsonism. From a neuropathological perspective, the correlate of FTD is frontotemporal lobar degeneration (FTLD) with different underlying proteinopathies (e.g. tau, TDP43, FET protein family). Though most FTD cases are idiopathic, there are genetic forms too. These are attributed to lesions in the *progranulin* and *C9ORF72* genes (both associated with TDP43-protein aggregates), but among the earliest genetic forms to be appreciated were mutations in the *MAPT* gene encoding the microtubule-associated protein tau [47, 58]. These FTLD-MAPT cases have proved of interest because of their potentially simple aetiology and their ability to be mobilized into model systems [43]. The P301L mutation found in FTLD–MAPT located in exon 10 is a case in point and affects just 4R tau isoforms (Fig. 1a). At the level of protein chemistry, the leucine substitution is suggested to increase conformational diversity of the tau polypeptide chain [34, 101].

Despite these foundational observations on FTD, there are still questions and layers of complexity pertaining to the FTLD–MAPT genetic sub-variety. Thus, curiously, the clinical heterogeneity encountered in the spectrum of FTD disorders does not disappear in its genetic forms, with heterogeneity of clinical presentation even observed within patients carrying the exact same *MAPT* P301L mutation [6, 32, 65, 103, 108]. Another conundrum posed by missense mutations such as P301L is that, while *MAPT* is widely expressed in the developing and adult brain [46, 106], disease may not become apparent until the fourth decade [97].

One avenue to gain purchase on these issues is to consider prion phenomena. The discovery that prion proteins (deriving from a precursor, the constitutively expressed cellular isoform of the prion protein, PrP^C^) can self-replicate in the absence of information encoded in nucleic acid sequences represented a new biological paradigm. This protein-only model is supported by multitude of evidence from biochemical, genetic, and animal studies [18, 20, 67, 75, 76], by in vitro synthesis of mice and hamster prions [4, 17, 26, 37, 53, 59, 110], and by generation of the synthetic human prions from recombinant protein expressed in bacteria [52]. Moreover, the early conceptual advance that prion phenomena are neither dependent upon viral-like nucleic acid genomes nor relegated to the realm of microbiology begged the question of applicability to other paradigms; Ure2 and Sup35 yeast prions both validated this extrapolation to proteins apart from PrP and provide two examples based on cytoplasmic proteins [39, 113]. For tau, rapidly expanding data accumulated in the cell and transgenic mice experiments suggest that different tau aggregates generated in vitro or in vivo can replicate in cells, accelerate and propagate the formation of tau aggregates in transgenics, and thus suggest that a prion-like replication phenomenon is taking place [48, 95].

In prion prototypes based upon PrP the misfolded form was commonly referred to as PrP^Sc^, the scrapie/pathologic from of PrP, but a subsequent insight was that a variety of alternative misfolded forms could account for “strain” phenomena that had already been deduced from in vivo infection experiments [12]. In the case of human prion strains, deriving from the study of Creutzfeldt-Jakob disease, the generally accepted definition and differentiation of strains is based on four necessary characteristics: (i) distinct clinical phenotype in the original host, (ii) distinct neuropathological characteristics, (iii) transmissibility and serial passage of the unique clinicopathological phenotype in experimental animals or cells expressing physiological levels of wild type PrP^C^, and (iv) differential conformational characteristics of prion protein determined with biophysical tools [52, 61]. While the extent to which data from tauopathy models and in vitro-generated tau aggregates fulfill these criteria is vigorously debated, we have explored these concepts to arrive at an understanding of the heterogeneity present within FTLD-MAPT. For this purpose we have used congenic lines of transgenic (*T*g) mice expressing low levels of a P301L mutant version of the 2N4R spliced form of human tau [29, 69] (Fig. 1a, b). Use of novel physicochemical tools to explore protein misfolding in these Tg lines and in brain tissue from human FTLD-MAPT-P301L patients has provided a number of unexpected insights, as described below.

## Material and methods

### Ethics statement

Ethical review at the University of Alberta was performed by the Research Ethics Management Office, protocols AUP00000356 and Pro00079472. All other procedures were performed under protocols approved by the Institutional Review Board at Case Western Reserve University (USA) and at the IDIBAPS brain bank (Barcelona, Spain). In all cases, written informed consent for research was obtained from patients or legal guardians and the material used had appropriate ethical approval for use in this project. All patients’ data and samples were coded and handled according to NIH guidelines to protect patients’ identities.

### Human brain samples

FTLD-MAPT-P301L patients of both sexes were as described previously [7] and as per Table 1. Clinical features of the patients were assessed as per contemporaneous criteria for diagnosis [42, 80]. Coronal sections of human brain tissues were obtained at autopsy and stored at − 80 °C. Three 200–350 mg cuts of frontal cortex (superior and more posterior middle gyri) and cerebellar hemisphere were taken from each brain and used for molecular analyses. The other symmetric cerebral hemisphere was fixed in formalin and processed for histologic and immunohistochemical purposes. Control brain samples were obtained from patients who died from non-neurological diseases; diagnostic neuropathology and retrospective chart reviews were carried out for all subjects, with particular attention to ruling out other age-related neurodegenerative diseases [22, 33, 38, 78].

**Table 1:** Clinical and molecular characteristics of ten Iberian FTLD-MAPT-P301L cases

Case	Sex	Age at death	Duration (years)	Signs at onset	Initial diagnosis	MAPT haplotype	ApoE	PMI (hrs)	Frontal cortex	Dentate nucleus of the cerebellum		Hippocampus		Parahippocampus
									Neuronal loss/AT8	*D*/*N*	Neuronal loss/AT8	*D*/*N*	Neuronal loss/AT8	Neuronal loss/AT8
1	M	52	6	Behavior	bvFTD	H1/H1	e3/e3	13.5	++/+++	3.6 ± 0.2	−/−	1.0 ± 0.0	−/+++	++/+++
2	M	58	5	Behavior	bvFTD	H1/H1	e3/e4	13	+++/+++	4.1 ± 0.1	+/-	1.0 ± 0.1	−/+++	++/+++
8	M	58	7	Behavior	bvFTD	H1/H1	e3/e4	10	+++/++	4.9 ± 0.3	−/+	1.0± 0.1	−/+++	+++/+++
7^a^	F	61	5	Behavior	bvFTD	H1/H1	e3/e3	14.8	++/+++	4.6 ± 0.1	−/+	1.7 ± 0.1	−/+++	++/++
4	M	72	13	Memory	AD	H1/H1	e3/e3	5.8	+++/+++	3.7 ± 0.3	−/+	1.0 ± 0.1	+/+++	+++/+++
6	M	53	7	Memory	AD	H1/H1	e3/e3	16.7	+++/+++	12.6 ± 1.2	−/−	1.2 ± 0.1	−/+++	+++/+++
9	M	75	7	Behavior	AD	H1/H2	e3/e3	5.9	++/+++	5.9 ± 0.1	−/−	3. ± 0.1	−/+++	++/+++
5	F	63	4	Language	AD or svPPA	H1/H2	e3/e3	7.3	++/++	4.6 ± 0.1	−/+	1.2 ± 0.2	−/+++	++/+++
3	M	56	10	Language	svPPA	H1/H1	e3/e3	6.3	+++/+++	10.3 ± 0.9	+/−	1.0 ± 0.1	−/+++	+++/+++
12	M	49	6	Language	svPPA	H1/H1	e3/e3	7.4	++/+++	8.2 ± 0.5	−/+	3.3 ± 0.1	ND	ND

Table after [7], arranged by clinical diagnosis: *bvFTD* behavioral variant of FTD, *svPPA* semantic variant of primary progressive aphasia, *AD* Alzheimer disease, *PMI* post mortem interval. All presented cases were FUS-negative, TDP43-negative. *D*/*N* denatured/native ratio in CDI assay, ± standard deviation

Age at onset was 51.3 ± 4, 60.7 ± 11.9, 50.0 ± 7 years for bvFTD, AD and svPPA, respectively. Age at death was 57.3 ± 3.8, 66.7 ± 11.9 and 56.0 ± 7 years, respectively. None of the differences in ages between the different initial diagnoses were significant

^a^Also features globular glial tauopathy affecting oligodendrocytes

**Table 2:** Percent distribution, average widths and period of helicity of each fibril morphology from CSA Type 2, 3 and 4 mice

	Type 2	Type 3	Type 4
% Straight fibrils	24.6%	87.0%	96.2%
Average width (nm)	22.47 ± 2.97	19.53 ± 3.45	19.24 ± 2.93
Average period of helicity (nm)	–	–	–
% Coiled fibrils	44.6%	1.6%	–
Average width (nm)	22.77 ± 1.83	20.43 ± 1.80	–
Average period of helicity (nm)	95.85 ± 9.39	92.59 ± 3.94	–
% Twisted ribbon-like fibrils	12.3%	1.3%	–
Average width (nm)	19.96 ± 1.46	23.65 ± 3.39	–
Average period of helicity (nm)	146.12 ± 5.86	134.97 ± 8.47	–
% Other	16.9%	10.1%	3.8%

The distribution of each fibril morphology was calculated from the fibril counts. Type 2 samples contain all three fibril morphologies, with coiled fibrils being the predominant form (*n* = 65). Type 3 samples contain a high proportion of straight fibrils, although coiled and twisted ribbon fibrils are still present (*n* = 553). Type 4 samples are exclusively composed of straight fibrils (*n* = 598). ‘Other’ refers to fibrils which were obscured or did not fit into the other morphologies. The width of fibrils were measured and found to be consistent across all fibril morphology regardless of CSA Type. In contrast, the period of helicity differed between twisted ribbon-like and coiled fibrils, with twisted ribbon-like fibrils having a longer helical period. Helicity measurements were not possible for straight fibrils as they do not display a visible crossover region. Width and helicity measurements were made in Adobe Photoshop using the ‘Ruler’ tool

### Preparation of brain homogenates for conformational stability assay (CSA) and conformation-dependent immunoassay (CDI)

Slices of tissue weighing 200–350 mg were first homogenized to a final 15% (w/v) concentration in calcium-free and magnesium-free PBS, pH 7.4, by 3 × 75 s cycles with a Mini-Beadbeater 16 Cell Disrupter (Biospec). Homogenates were then diluted to a final 10% (w/v) in 1% (v/v) sarkosyl in PBS, pH 7.4 and re-homogenized. After clarification at 500×*g* for 5 min, additional sarkosyl was added to a final 2% and the samples were spun at 15,500 rpm at 4 °C for 30 min in Allegra X-22R tabletop centrifuge (Beckman Coulter). To investigate protease-sensitive and resistant fractions of tau, the sample aliquots were digested with Proteinase K (PK) according the rodent and human prion protocol at 1:50 enzyme/total protein (weight/weight) ratio [50, 85, 87]. The pellet containing sarkosyl-insoluble tau protein was resuspended in PBS, pH 7.4 and divided into two aliquots: the first one was incubated with 100 µg/ml of PK (Amresco) for 1 h at 37 °C with shaking of 600 rpm in an Eppendorf Thermomixer (Eppendorf) and the second one, untreated, was mixed with protease inhibitors cocktail (0.5 mM PMSF and aprotinin and leupeptin at 5 µg/ml, respectively). After blocking PK-treated aliquots with a protease inhibitor cocktail, samples were stored for analysis at − 80 °C.

### Sandwich-formatted CDI for tau strains

The CDI for human tau was performed as described previously for mammalian prions [19, 50, 85, 87] with the following adaptations: First, we used white Lumitrac 600 High Binding Plates (E&K Scientific) coated with monoclonal antibody (mAb) DA9 (epitope 102–139, gift of Dr. Peter Davies) in 200 mM NaH_2_PO_4_ containing 0.03% (w/v) NaN_3_, pH 7.5. Each sample was split in two aliquots: the first one was denatured (D) with final concentration of 4 M Gdn HCl and the second one, native (N), was untreated. Aliquots of 20 µl from each fraction containing 0.007% (v/v) of Patent Blue V (Sigma) were directly loaded into wells of white strip plates prefilled with 200 µl of casein/0.05% Tween® 20 in TBS, pH 7.4 (SurModics). Finally, the captured tau was detected by a europium-conjugated [85] anti-tau mAb 77G7 (epitope 316–355; Biolegend) and the time-resolved fluorescence (TRF) signals were measured by the multi-mode microplate reader PHERAstar Plus (BMG LabTech). The recombinant 2N4R splicing variant of human tau (tau441) expressed in *E. coli* and purified as described [27] was used as a calibrant after complete denaturation in 4 M Gdn HCl. The initial concentration of recombinant human tau441 was calculated from the absorbance at 280 nm and molar extinction coefficient 7450 M^−1^ cm^−1^. The purified recombinant proteins were dissolved in 4 M Gdn HCl and 50% Stabilcoat (SurModics), and stored at − 80 °C. The concentration of tau was calculated from the CDI signal of denatured samples using serially-diluted tau441 to create a calibration curve. The TRF signal of denatured and native sample aliquots is expressed as a ratio (D/N) and is a measure of exposed epitopes in the native state against the reference of fully unfolded protein.

### Monitoring dissociation and unfolding of tau strains by CSA

The sequential denaturation of human tau was performed as described previously for mammalian prions [50, 85], with the following modifications: Frozen aliquots of samples containing tau were thawed, sonicated 3 × 5 s at 80% power with Sonicator 4000 (Qsonica), and the concentration was adjusted to a constant ~ 200 ng/ml of tau. 15 µl aliquots in 15 tubes were treated with increasing concentrations of 8 M Gdn HCl containing 0.007% (v/v) Patent Blue V (Sigma) in 0.25 M or 0.5 M increments. After 30 min incubation at room temperature, individual samples were rapidly diluted with casein/0.05% Tween® 20 in TBS, pH 7.4 (SurModics) containing diminishing concentrations of 8 M Gdn HCl, so that the final concentration in all samples was 0.411 M. Each individual aliquot was immediately loaded to dry white Lumitrac 600, High Binding Plates (E&K Scientific), coated with mAb DA9 previously blocked with casein/0.05% Tween® 20/6% sorbitol/0.03% sodium azide, and developed in accordance with CDI protocol using europium-labeled mAb 77G7 for detection as described for mammalian prions [50, 85, 87–89]. The raw TRF signal was converted into the apparent *F*_app_ as follows [50]: *F*_app_ = (TRF_OBS_ − TRF_N_)/(TRF_U_ − TRF_N_) where TRF_OBS_ is the observed TRF value, and TRF_N_ and TRF_U_ are the TRF values for native and unfolded forms, respectively, at the given Gdn HCl concentration [84]. To determine the concentration of Gdn HCl where 50% of tau is unfolded [(Gdn HCl)_1/2_], the data were fitted by least square method with a sigmoidal transition model (Eq. 1):

1 $$F_{{{\text{app}}}} = F_{0} + \frac{{\left( {F_{{\max }} - F} \right)_{0} }}{{1 + e^{{\{ (c_{{1/2}} - c)/r\} }} }}$$

The apparent *F*_app_ in the TRF signal is the function of Gdn HCl concentration(c); c_1/2_ is the concentration of Gdn HCl at which 50% of tau strains is dissociated/unfolded and *r* is the slope constant.

### Direct format of CSA for protease-resistant core of tau strains

For the PK-resistant misfolded aggregates of tau, frozen aliquots of PK-treated tau were thawed, sonicated 3 × 5 s at 80% power with Sonicator 4000 (Qsonica), and the concentration was adjusted to constant ~ 200 ng/ml of tau. 15 µl aliquots in 15 tubes were treated with increasing concentrations of 8 M Gdn HCl in 0.25 M or 0.5 M increments. After 30 min incubation at room temperature, individual tubes were rapidly diluted with H_2_O containing diminishing concentrations of 8 M Gdn HCl so that the final concentration in all samples was 0.2 M. Each aliquot was immediately loaded in triplicates to dry white Lumitrac 600, High Binding Plates (E&K Scientific). Following incubation at 4 °C and blocking with casein/0.05% Tween® 20/6% sorbitol, the plates were developed with europium-labeled mAb 77G7. The raw time-resolved fluorescence (TRF) signals obtained with the multi-mode microplate reader PHERAstar Plus (BMG LabTech) were converted into the apparent *F*_app_ and to obtain the concentration of Gdn HCl where 50% of tau is unfolded [(Gdn HCl)_1/2_], the data were fitted by least square method with a sigmoidal transition model as described for sandwich CSA.

To deconvolute the non-sigmoidal denaturation profiles, we used statistical mechanical deconvolution and Gaussian models originally developed for proteins that undergo more than one-step thermal denaturation [91]. The Gaussian model was also used to analyze the fractional change after PK: the CSA obtained after PK treatment were subtracted from *F*_app_ values obtained before PK (Δ*F*_app_ = *F*^0^ − *F*^PK^) and then fitted with a Gaussian model to estimate the proportion and average stability of protease-sensitive tau strains conformers.

2 $$\Delta F_{{{\text{app}}}} = F_{0} + A^{{\left\{ {\left( { - c - c_{0} } \right)^{2} } \right\}}}$$

In this model, the PK-induced fractional change is Δ*F*_app_, *F*_0_ is fractional change at 0 concentration of Gdn HCl, and c_0_ is the Gdn HCl concentration at the maximum height *A* of the peak [50].

### Statistical analysis for CSA and CDI

We investigated the effect of concentration and stability of pathogenic tau strains in Gdn HCl before and after PK treatment [85] on clinicopathological phenotype and duration of the disease in cases of FTLDs associated with *MAPT* P301L mutation and in TgTau^P301L^ mice. In the comparisons of different groups, p-values were calculated using ANOVA and unfolding curves of tau among different mice and different clinical phenotypes in FTLD-MAPT-P301L cases were compared by the log rank (Mantel-Cox) and generalized Wilcoxon test. All the statistical analyses including regression modeling were performed using SPSS 25 software (Statistical Package for Social Sciences, SPSS Inc. Chicago, IL).

### Transgenic mice

TgTau^P301L^ mice and their non-Tg littermates were derived by injections into oocytes from 129/SvEvTac x FVB/NJ F_1_ mice and then bred further to obtain congenic and incipient congenic derivatives as described previously [29, 69]. Animals were maintained in ventilated racks (Tecniplast) and fed irradiated chow (LabDiets, 5053). They were housed with a 12 h/12 h light/dark cycle. Cage environmental enrichment comprised 5 cm diameter plastic tubes and nesting material ("Nestlets", Ancare Inc.). For routine genotyping of litters, forward primer 5′-TGGATCTTAGCAACGTCCAGTCC-3′ and reverse primer 1587 = 5′- CTCTCCTCTCCACAATTATTGACCG-3′ were used to amplify tail-derived genomic DNA. Polymerase chain reaction (PCR) cycle conditions were 94 °C 3 min then (94 °C 20 s, 55 °C 20 s, 72 °C 30 s) × 35, 72 °C for 7 min, then a 4 °C hold, overall yielding a diagnostic fragment of 521 bp. An equal number of *T*g animals of both sexes were assigned to the experiment and allowed to age to the dates indicated herein. Immunohistochemistry to assign pathology classes was performed as described previously [29]. All animal experiments were performed in accordance with local and Canadian Council on Animal Care ethics guidelines.

### Nested PCR for detecting integrated MAPT cDNAs in brain genomic DNA

Nested PCR with a short-chain extension time was performed using primers on either side of a > 10 kb intron such that amplification products would only be obtained if reverse transcripts of the *MAPT* transgene were integrated into the genomic DNA of brain tissue. The primary PCR reaction mixture was 100 ng of genomic DNA extracted from brain homogenate, 10 pmol of primers (forward primer: 5′-GAGAAGGCACATCGAGTCCA-3′ in the hamster PrP exon 1 and reverse primer: 5′-GTTCTCAGTGGAGCCGATCT-3′ in the *MAPT* coding region), 80 µM dNTPs (Invitrogen), 1.25 U recombinant Taq DNA polymerase (Invitrogen), 1 × PCR Buffer (Invitrogen) and 1.5 mM MgCl_2_ in a final volume of 25 µl. Reaction conditions were as follows: 94 °C, 30 s (94 °C, 30 s; 52 °C, 30 s; 72 °C, 1 min) for 40 cycles. Nested PCR was performed using 1 µl of a 1/500 dilution of the primary PCR as template using 10 pmol of primers (forward: 5′-CTCGTCGCGTCGGTGGCA-3′, reverse: 5′- TGCGATCCCCTGATTTTGGAGG -3′) 80 µM dNTPs (Invitrogen), 1.25 U recombinant Taq DNA polymerase (Invitrogen), 1 × PCR Buffer (Invitrogen) and 1.5 mM MgCl_2_ in a final volume of 25 µl. Reaction conditions were: 94 °C, 30 s (94 °C, 30 s; 55 °C, 30 s; 72 °C, 1 min) for 40 cycles. The expected size of the reaction product is 624 bp. As a positive control and to determine the limiting number of DNA copies required for detectable amplification, a custom gene was synthesized (Integrated DNA Technologies) corresponding to the *MAPT* transgene cDNA. The reaction mixture and conditions were identical to above except that in addition to the 100 ng of genomic DNA in the primary PCR, a dilution series of the plasmid was also included (2.57 × 10^6^ to 2.57 copies).

### In situ hybridization on mouse brain tissue

In situ hybridization (ISH) was performed on 5 µm formalin-fixed paraffin-embedded tissue using the RNAscope® 2.5 High Definition (HD) detection Reagent (Advanced Cell Diagnostics, ACD). To circumvent potential cross-reactivity for mouse versus human *MAPT* transcripts, an ISH probe Mau-Prnp-No-XMm-O1 (#804,721) was custom designed; this recognizes a region of the Syrian Golden hamster PrP 3′ untranslated region (1049–1984 of GenBank accession XM_013112401.1) contained in the cos.Tet cosmid used to make the TgTau^P301L^ mouse line and does not cross-react with endogenous mouse *Prnp* transcripts. A probe targeting the 4-hydroxy-tetrahydrodipicolinate reductase (dapB) gene of bacteria was used as a negative control probe and a probe targeting Peptidyl-prolyl *cis*–*trans* isomerase B (Ppib) was used as positive control. The RNAscope® assay was performed according to the manufacturer’s protocol [109] with 15 min of target retrieval and 30 min of protease digestion. The slides were then analyzed using digital slide scanner NanoZoomer XR (Hamamatsu SZK).

### Extraction of insoluble tau protein

Fractions of mouse brain were prepared as previously described [92]. Briefly, tissues were homogenized in 10 volumes of Tris-buffered saline (TBS: 50 mM Tris/HCl (pH 7.4), 274 mM NaCl, 5 mM KCl) with 1% protease inhibitor cocktail (Roche), 1% phosphatase inhibitor cocktail (Roche) and 1 mM phenylmethylsulfonyl fluoride (PMSF). The homogenates were then centrifuged at 27,000×*g* for 20 min at 4 °C to obtain supernatant (“SUP1”) and pellet fractions. The pellet was then re-suspended in five volumes of high salt/sucrose buffer (0.8 M NaCl, 10% sucrose, 10 mM Tris/HCl, (pH 7.4), 1 mM EGTA, and 1 mM PMSF) and centrifuged at 27,000×*g* for 20 min at 4 °C. The supernatants obtained from this step were collected and incubated with sarkosyl (1% final concentration; Sigma) for 1 h at 37 °C, followed by centrifugation at 150,000×*g* for 1 h at 4 °C to obtain salt and sarkosyl-extractable (“SUP3”) and sarkosyl-insoluble (“P3”) fractions. The P3 pellet was resuspended in 50 µl TE buffer (10 mM Tris/HCl (pH 8.0), 1 mM EDTA).

### Western blots

Western blotting was performed as described previously [69, 105]. Samples were prepared in a loading buffer containing SDS and 2-mercaptoethanol and boiled for 10 min. They were then electrophoresed on a 10% Tris-tricine gels using a Bio-Rad system and transferred to polyvinyl difluoride (PVDF; Millipore) membranes (wet transfer) and blots were then blocked with 5% skim milk in 1xTBS-0.1% Tween® 20 for 1 h at room temperature and incubated with primary antibodies at 4 °C overnight. CP27 (detecting total human tau protein) antibody was used at 1:500 dilution, while ET3 (detecting 4R tau residues 273–288) was used at 1:250 dilution. Membranes were subsequently incubated with secondary antibody (Bio-Rad) at 1/10,000 for 1 h at room temperature and visualized using enhanced chemiluminescence (ECL, Pierce). Anti-actin antibody (Sigma) was used for quantification blots (1/2,000 dilution).

### Trypsin digestion of sarkosyl-insoluble tau extracts and in-gel analysis

Sarkosyl insoluble fractions (P3) were subjected to trypsin digestion as previously described [44, 104] with some modifications. Reactions were set up on 5 µg of protein from a P3 fraction depending on the protein concentration of the samples with sequencing grade trypsin (Pierce), with trypsin/protein ratio adjusted for 1/25 (unless stated otherwise). After 30 min of incubation at 37 °C, the reaction was stopped by addition of sample buffer and boiled for 10 min. Samples were western blotted using ET3 antibody. For in-gel digestion of sarkosyl-insoluble material, each lane of trypsin-digested P3 material was separated into strips. The samples were transferred to a round bottom 96-well plate and 150 µl of destaining solution (50 mM ammonium bicarbonate, 50% acetonitrile) was added into each well. The plate was incubated for 10 min at 37 °C. The solution was removed from the wells and the destaining step was repeated 3–4 times. The solution was removed and replaced by acetonitrile. The samples were incubated again at 37 °C until the gel bands became white. The remaining acetonitrile was removed, and the samples were dried at 37 °C for 10 min. The samples were rehydrated with 175 µl of reducing solution (100 mM ammonium bicarbonate and 5 mM β-mercaptoethanol) and incubated for 30 min at 37 °C. After that, the reducing solution was removed and 175 µl of alkylating solution (10 mg/ml of iodoacetamide and 100 mM ammonium bicarbonate) was added. Samples were incubated for 30 min at 37 °C. The gel bands were subsequently washed with 175 µl of 100 mM ammonium bicarbonate and incubated for 10 min at 37 °C. The samples were then incubated for 10 min at 37 °C in acetonitrile. Once the gel pieces became white, acetonitrile was removed, and the samples were dried at 37 °C for 10 min. The protein samples were then digested with 50 µl of digestion buffer (50 mM ammonium bicarbonate and 20 ng/µl of trypsin, Promega Inc.). The solutions containing tryptic peptides were transferred to a V-bottom 96-well plate. Tryptic peptides were further extracted from the gel with 2% acetonitrile and 1% formic acid followed by incubation at 37 °C for 1 h. The extraction was repeated using 50% acetonitrile and 0.5% formic acid followed by incubation at 37 °C for 1 h. The samples were freeze-dried under vacuum overnight. The peptides recovered from some of the wells were combined to generate a total of 8 fractions. The eight fractions covered between 250 and 75 kDa (F1), 75–60 kDa (F2), 60–50 kDa (F3), 50–37 kDa (F4), 37–25 kDa (F5), 25–20 kDa (F6), 20–15 kDa (F7) and 15–8 kDa (F8). The samples were re-suspended in 0.2% formic acid before analysis by LC–MS/MS.

### Mass spectrometry

Peptides were analyzed using a nanoflow-HPLC (Thermo Scientific EASY-nLC 1000 System) coupled to the Q-Exactive (Thermo Fisher Scientific) mass spectrometer. A trap column (5 μm, 100 Å, 100 μm × 2 cm, Acclaim PepMap 100 nanoViper C18; Thermo Fisher Scientific) and an analytical column (3 μm, 300 Å, 75 μm × 15 cm, PepMap RSLC C18; Thermo Fisher Scientific) were used for the reverse phase separation of the peptide mixture. Peptides were eluted over a linear gradient over the course of 90 min from 0 to 95% acetonitrile in 0.2% formic acid. Data analysis was performed using ProteinDiscoverer (v1.4.1.14) software against a tau mouse wt sequence and the tau P301L mutant (sequences downloaded from https://www.uniprot.org/uniprot/P10637). Search parameters included two missed trypsin cleavages, a precursor mass tolerance of 10 ppm, a fragment mass tolerance of 0.01 Da, carbamidomethylation of Cys (static modification) and oxidation of Met and deamidation of Asn and Gln (dynamic modifications). A decoy database search was performed to evaluate the false-positive rates. The strict target false discovery rate was set at 0.01 and the relaxed FDR was set at 0.05. Results reported include only the peptides identified at medium and high confidence.

### Tau cell seeding assay

HEK293 cells (which are derived from a female donor and lack a Y chromosome) stably expressing a YFP-tagged human tau 4R repeat domain (RD) fragment that includes aggregation-prone mutations (P301L/V337M, LM; pro-aggregation) ("HEK-tauRD-LM-YFP cells") [94] were plated in Dulbecco’s modified Eagle’s medium (Gibco) supplemented with 10% fetal bovine serum (HyClone) and 1% penicillin/streptomycin (Gibco) at 1 × 10^6^ cells/well in 12-well plates and grown at 37 °C, 5% CO_2_, in a humidified incubator. The next day, the cells were seeded with liposome–protein complexes derived from each class of TgTau^P301L^ mouse brain and human P301L patients. Mouse brain homogenate (5–8 mg/ml protein solution) or human brain homogenate (10 mg/ml protein solution) was combined with an equal volume of Lipofectamine 3000 (Thermo Fisher) and added into each well containing 1 ml of culture medium. TgTau^P301L^ samples were adjusted using tau concentrations derived from CDI measurements to obtain a total mass of 20 ng tau per well. The cells were then incubated for 6 h at 37 °C and the media containing the liposome-protein complex were replaced with fresh growth media. The cells were subcultured into glass cover-slips double-coated with poly-d-lysine and laminin. At 6 days after seeding, images of cells were acquired using a confocal microscope, ZEN Digital Imaging for LSM 700 (laser scanning microscope, Zeiss, Jena, Germany) and analyzed with Zen 2010b SP1 imaging software (Zeiss) and Image J (https://imagej.nih.gov/ij/). The frequency of tau inclusion morphologies and cells seeding efficiencies were determined by analyzing at least 1000 cells from 6 different areas of the cover slip for each treatment; three blinded observers were used to score morphologies with data presented as arithmetic averages.

### Negative stain electron microscopy

Aliquots (5 μl of 0.5 mg/ml protein solution) of sarkosyl-insoluble P3 fractions or CSA samples were loaded onto freshly glow-discharged 400 mesh carbon coated copper grids (Electron Microscopy Sciences) and adsorbed for ~ 1 min. Next, the grids were sequentially washed with 50 μl each of 0.1 M and 0.01 M ammonium acetate and negatively stained with 2 × 50 μl of freshly filtered 2% uranyl acetate. After drying, the grids were examined with a Tecnai G20 transmission electron microscope (FEI Company) using an acceleration voltage of 200 kV. Electron micrographs were recorded with an Eagle 4 k × 4 k CCD camera (FEI Company). Morphologies of individual tau filaments were classified into "straight filaments", "coiled filaments", and "twisted ribbon-like filaments" as described previously [29].

### Data analysis

Departures from normal distribution were checked using the Kolmogorov–Smirnov (K–S) goodness of fit test. A general linear model of factorial ANOVA (Statistical Package for Social Sciences, SPSS v.22, Inc. Chicago), with genotype, line/genetic background as between subject factors, or repeated measures analysis of variance (RMANOVA) with the type of cell count (total cell and neuronal counts) as within subject factors were used to analyze the data. Eta squared (*η*^2^) was used to estimate the effect size, i.e. the proportion of variance associated with each of the main effects and interactions. Bonferroni adjustment of α level (MODLSD Bonferroni *t*-tests, SPSS v22) was applied in multiple planned comparisons. In the case when data represented discrete category measures on a nominal scale and did not meet the assumption of parametric statistics, a *χ*2 test of independence was used to test for homogeneity between the groups.

## Results

### Phenotypic diversity and protein misfolding

Cohorts of congenic derivatives of a TgTau^P301L^ mouse line totaling 243 animals were profiled for tau deposition with the AT8 antibody as used for Braak staging of neurofibrillary pathology in human material [8] (Supplementary Table 1). The finding that several categories of pathological deposition of tau were represented in both sexes in *T*g mouse stocks maintained in independent C57BL/6Tac, 129SvEv/Tac and FVB/NJ inbred sublines extends and underscores earlier analyses and argues against genomic variation in driving this diversity [29]. Concerning chronology, we considered whether different classes of neuroanatomical distribution of tau merely reflected pathological stages in a predictable progression. In animals with notable frontal deposition of tau, the age differences for classes I vs. II vs. III of pathology did not reach significance (Supplementary Table 1). Mice scored with brain stem deposition (class IV pathology) were younger than classes I, II and III mice (*P* = 0.03, 0.0006, and 0.0004, respectively) in the C57BL/6Tac background, with a similar trend in 129SvEv/Tac and FVB/NJ TgTau^P301L^ mice for class scoring III vs. class IV pathology (P = 0.011 and 0.007, respectively).

With regard to transgene expression as a variable, in situ hybridization for *MAPT* transcripts originating from the autosomally-inherited transgene did not reveal variegation but instead pan-neuronal expression (Fig. 1b), as anticipated from previous use of prion promoter constructs. Given recent interest in somatic mutational events leading to diversity in gene expression by integration of mRNA reverse transcripts [57], we sought evidence for these types of genomic rearrangements. This experiment used a configuration of nested primers for PCR of genomic DNA retrieved from brains of 27 aged TgTau^P301L^ mice; with a sensitivity of approximately 1/1300 below the level of a single-copy gene present in a haploid genome size estimated at 3 pg, we were unable to find evidence for extraneous cDNA copies of tau coding sequences in any of these aged TgTau^P301L^ mice (Supplementary Fig. 1).

With data failing to support varying transcript expression as an underlying driver of heterogeneity (and noting a limited relevance of mRNA expression on pathogenesis inferred for rTg4510-P301L mice [36]), we sought a different explanation. We considered this process in terms of a homogeneous pool of physiologically-folded tau precursor leading to different species of misfolded tau (i.e. different conformers) and derived corresponding conformation-dependent immunoassays (CDIs; [85]) and conformational stability assays (CSAs) to appraise abnormally folded tau. In the first procedure tau is first exposed to the denaturant guanidine hydrochloride (Gdn HCl) and then exposed to europium-labeled mAb against epitopes that are otherwise hidden under native conditions [85] (Fig. 1c, d; Supplementary Fig. 2). The second procedure involves the use of multiple Gdn HCl concentrations to progressively increase denaturation and corresponding differences in stability profiles have provided evidence for distinct conformations of prion and Aß strains [22, 45, 50, 51, 85, 90]. Importantly, CDI ratios and CSA unfolding conformational signatures are independent of the absolute concentrations of the misfolded species and the procedure does not involve purification or an in vitro amplification step before detection—a process that can alter the in vivo conformational repertoire and biological properties of strain isolates [2, 52]). Notwithstanding data for human and animal-adapted prions indicating that CSAs differentiate strains regardless of posttranslational modifications such as complex glycosylation and glycolipidation [45, 51, 52, 86, 90], we selected the antibody for conformational monitoring with an epitope in the R3/4 boundary of microtubule binding repeats, an area of tau less decorated by posttranslational modifications (Fig. 1c) [13, 62, 100]. Capture DA9 and Europium-labeled detection 77G7 monoclonal antibodies used in these assays have linear (not conformational) epitopes and detect only open (unfolded) conformers of monomeric tau as outlined in Fig. 1 and demonstrated in Fig. S2 with monomers and fibrils generated from recombinant tau441 protein in vitro. Both antibodies have epitopes outside known phosphorylation sites in PHFs and acetylated Lys174, Lys274 and Lys280 that have been described in the tau deposits of AD, FTDP-17, and PiD patients [1, 2, 8].

### CDI of human tau

Both direct- and sandwich-CDIs performed against human Tau441 demonstrated a broad linear range (Supplementary Fig. 2). We assessed a set of human brain samples obtained from twelve FTD cases linked to a P301L *MAPT* mutation founder effect [7, 72] (Table 1). The cases were initially assigned clinical diagnosis of bvFTD, svPPA, and AD using contemporaneous clinical diagnostic criteria [42, 80] and with a final diagnosis of FTLD-MAPT-P301L based on *MAPT* gene sequencing and comprehensive neuropathological investigation of multiple brain areas. CDI for amyloid beta (Aß) and immunohistochemistry for Aß, alpha-synuclein, TDP-43, and FUS excluded coexistent proteinopathies and other comorbidities [22, 56, 83] (Supplementary Table 2, supplementary Fig. 3). Noting these data, the subset of bvFTD cases with predominant memory deficits leading to the initial clinical diagnosis of AD were assigned as a bvFTD sub-variety and designated as bvFTD*.

**Fig. 2:**
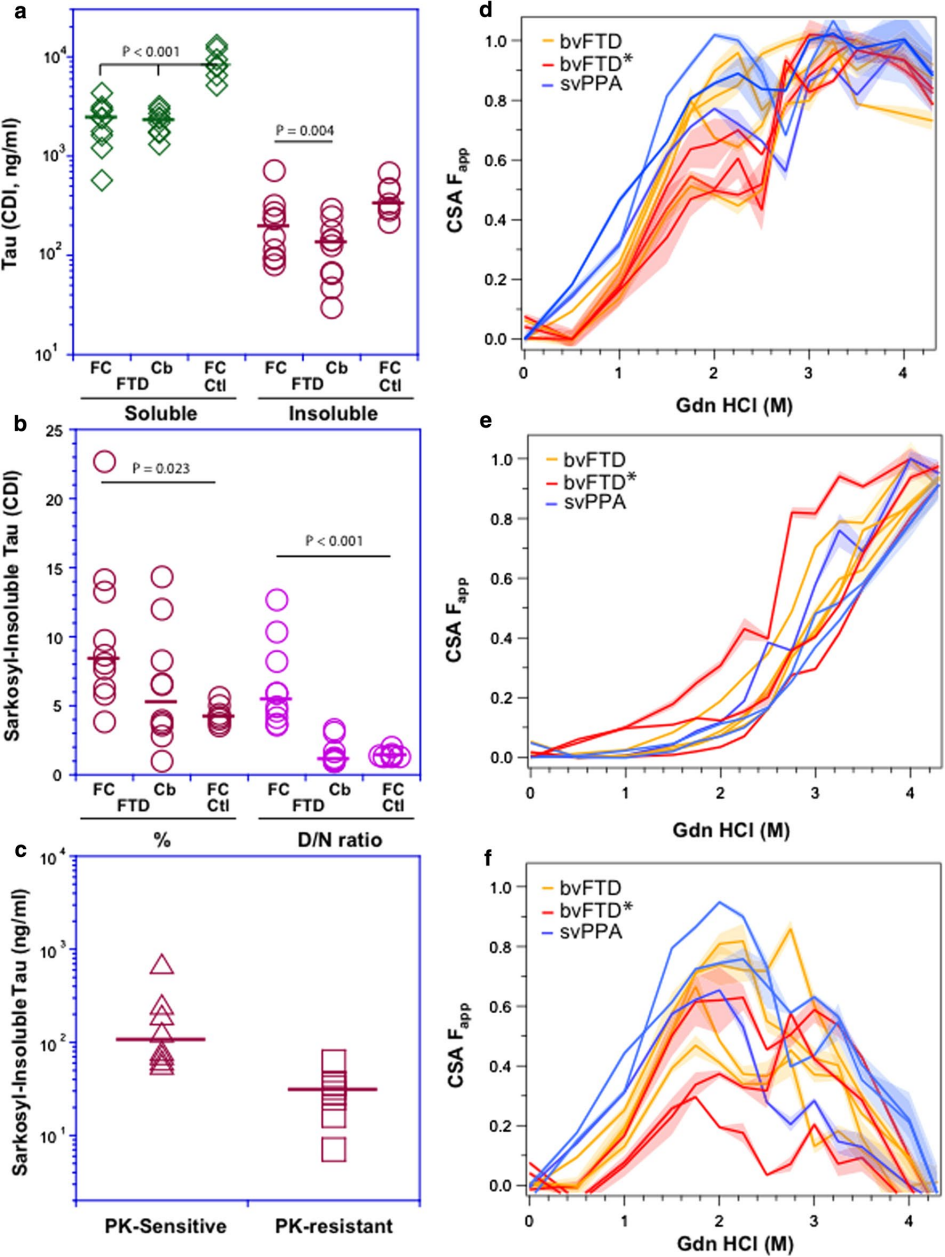
Levels and conformational profiling of tau in the brain tissue of FTLD-MAPT-P301L patients and age-matched controls. **a** Concentration of sarkosyl-soluble and insoluble tau protein in the frontal cortex (FC) and cerebellum (Cb) of FTLD cases and in the frontal cortex of age-matched non-neurological controls (FCctl). **b** Fraction (%) and conformation (denatured/native CDI signal ratio) of sarkosyl-insoluble tau in the frontal cortex (FC) and cerebellum (Cb) of FTLD cases, and cortex of non-neurological controls (FCctl). **c** Concentrations of Proteinase K-sensitive and resistant detergent-insoluble tau in the frontal cortex of FTLD patients. The diverse conformational profiles of sarkosyl-insoluble tau in different clinical phenotypes of FTLD-MAPT-P301L cases before (**d**) and after protease treatment (**e**), and differential curves for protease-sensitive tau conformers (**f**). The CDI measurements were performed with sandwich-formatted (before) and direct (after PK treatment) to monitor the concentration and conformation of R3,4 tau domain (residues 316–355) in native (N) and denatured (D) state after unfolding with 4 M Gdn HCl. Concentrations are an average of three independent measurements expressed in ng/ml of 10% brain homogenate; horizontal lines in plots indicate median. The values of F_app_ of each brain sample from native to denatured state are mean ± SEM obtained from triplicate measurements at each concentration of denaturant (Gdn HCl) and the SEM is depicted as a shade. Curve analysis was performed with nonlinear regression and the statistical significance was determined with generalized Wilcoxon test

We examined frontal cortex and a less affected region, the dentate nucleus of the cerebellum [7]). The detergent-soluble tau in the frontal cortex and cerebellum of FTD patients—FC(P301L) and Cb(P301L)—were ~ threefold lower than in frontal cortex of age-matched human non-neurological controls, FC (Ctl) (Fig. 2a), indicating a mild down-regulation effect. Interestingly, considering this as a likely cellular defense mechanism against pathogenesis, we observed a uniform down-regulation in normal (cellular) prion protein during prion infection [63] and low net tau has also been reported in brain cortex of AD cases [10, 68]. Concentrations of sarkosyl-insoluble aggregates of tau protein were more varied in patients than in controls and represented 4–23% and 1–14% of total tau in the frontal cortex and cerebellum, respectively, of individual FTD cases, reaching significance only for frontal cortex of FTLD-MAPT-P301L cases (Fig. 2b). In contrast to denatured/ native (D/N) signal ratios of ~ 1.0 in controls, these indicating little change in epitope availability after treatment with chemical denaturant, *D*/*N* values > 3 for the frontal cortex of FTLD-MAPT-P301L cases indicated tau species with a hidden R3, R4 domain before denaturation. The wide range of *D*/*N* values varying between 3.6 and 12.6 indicate a spectrum of distinct conformers (Fig. 2b; Table 1). Approximately 50% of these misfolded detergent-insoluble tau conformers were protease-sensitive (Fig. 2c). Two FTLD-MAPT-P301L cases also had D/N values above controls in the dentate nucleus of cerebellum (Table 1). Cumulatively, the CDI data indicate that individual FTLD-MAPT-P301L patients accumulate partially protease-sensitive, detergent-insoluble misfolded tau protein with distinct conformations, this accumulation being predominantly in the frontal cortex.

### Conformational diversity of tau in FTLD-MAPT-P301L patients

Frontal cortex samples of individual patients yielded complex CSA profiles with 50% of tau conformers (i.e. fractional change of unfolding (*F*_app_) values of 0.5) unfolding between 1 and 2.3 M Gdn HCl, multiple peaks at ~ 2 M Gdn HCl, followed by complete unfolding above 3 M Gdn HCl (Fig. 2d). PK treatment of detergent-insoluble tau drastically reduced the complexity of the unfolding profile and shifted the resulting sigmoidal curves and their midpoints to the higher concentrations of Gdn HCl between 2.2 and 3.2 M Gdn HCl (Fig. 2e). Performing a subtraction function on the individual curves from the original curves without PK treatment yielded the unfolding profiles for protease-sensitive misfolded tau (Fig. 2f). The dip between 2 and 3 M Gdn HCl in some profiles, which disappears after PK treatment, suggests rapid re-aggregation of misfolded protease sensitive tau conformers during the sample processing. Thus detergent-insoluble tau aggregates accumulating in the frontal cortex of FTLD-MAPT-P301L cases encompass a spectrum of distinct conformers within each individual case and with the majority of species being protease-sensitive (as per the mouse profiles). That the residual protease-resistant tau species did not converge to a single overlapping signature corroborates the conformational diversity of protease-sensitive tau conformers. At the level of neuropathological examination, these brains show a predominance of “pretangle” type pathology in neurons and rarely fibrillar aggregates as seen in AD [7].

### CDI of mouse brain samples

**Fig. 3:**
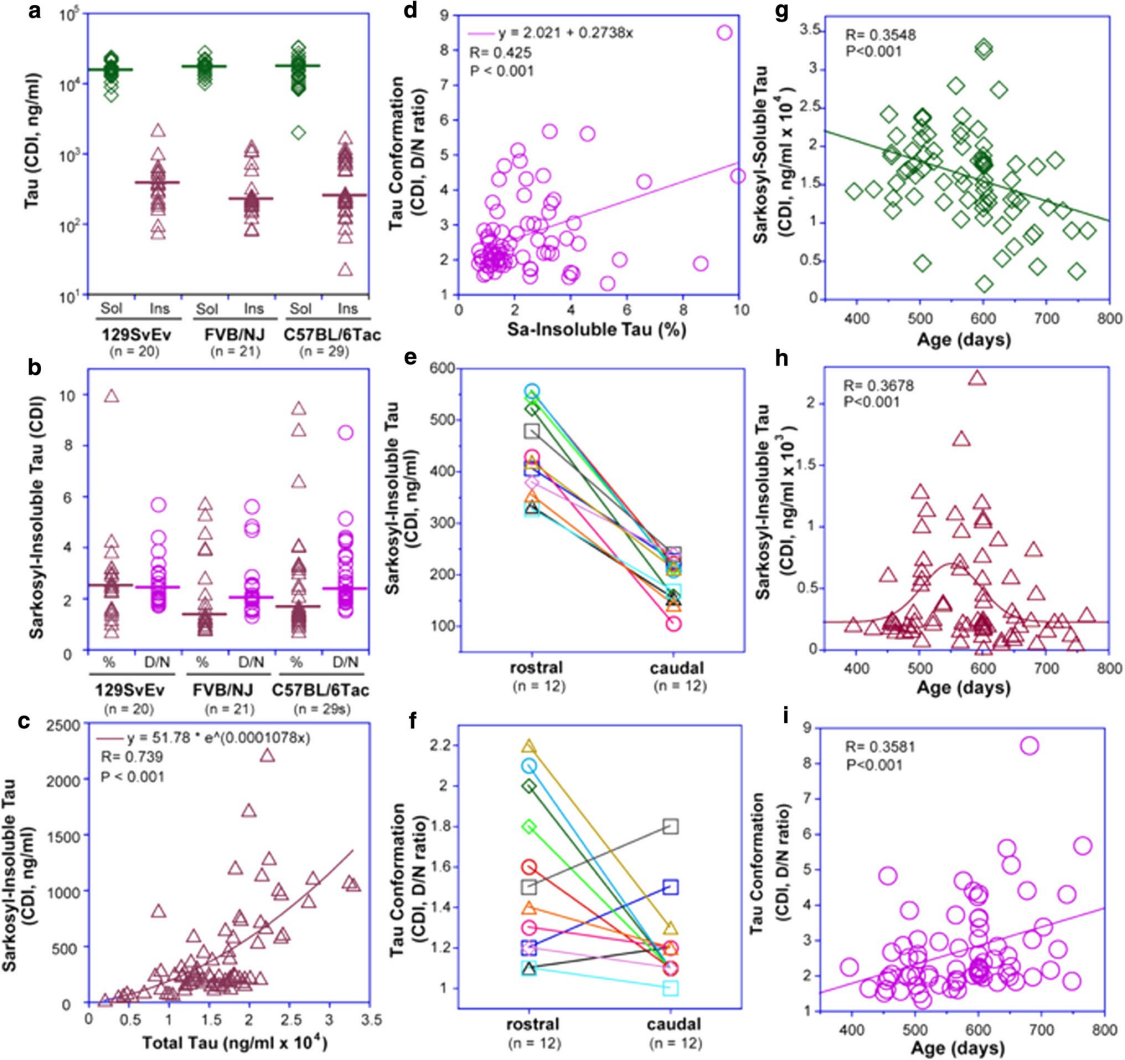
Levels, conformational characteristics, localization and age evolution of misfolded tau in TgTau^P301L^ mice. **a** Inter-individual variability of sarkosyl-soluble (Sol) and sarkosyl-insoluble (Ins) tau in TgTau^P301L^ mice with different backgrounds; (**b**) fraction (% of total) and denatured/native (*D*/*N*) ratios of sarkosyl-insoluble brain tau; horizontal lines in (**a**) and (**b**) indicate median. **c** Exponential regression model of dependency of sarkosyl-insoluble tau on total tau levels; (**d**) *D*/*N* ratios correlate with increasing concentrations of sarkosyl-insoluble tau. **e**, **f** Rostro-caudal brain gradients for levels and *D*/*N* ratio of sarkosyl-insoluble tau as assessed for 12 aged TgTau^P301L^ mice. **g** Age dependency of sarkosyl-soluble tau; (**h**) accumulation of detergent insoluble tau aggregates, and (**i**) conformational diversity of detergent-insoluble tau in TgTau^P301L^ mice revealed by *D*/*N* ratios. Brain samples were measured with CDI in triplicates as described in Fig. 4 and the data are fitted with exponential (**c**), linear (**g**, **i**) or Gaussian distribution (**h**) model

Next, concentrations of sarkosyl-soluble tau in mouse hemi-brain samples divided along a sagittal axis and profiled by CDI were similar across all three genetic backgrounds (Fig. 3a), compatible with prior blot analyses [29]. The ~ twofold higher levels than in non-transgenic controls reflect the differential affinity of capture antibody toward mice and human tau. Levels of sarkosyl-insoluble tau were more variable in C57BL/6Tac mice than in other inbred lines and reached ~ twofold higher tau variance, kurtosis and positive skewness towards a higher frequency of insoluble tau aggregates (Fig. 3a). CDI denatured/native (*D*/*N*) ratios on the same samples demonstrated a similar trend, i.e. higher *D*/*N* values in C57BL/6Tac mice, suggesting a higher degree of tau misfolding events than in comparable 129SvEv/Tac and FVB/NJ *T*g mice (Fig. 3b). Regression analysis shows a significant correlation between total and insoluble tau levels (Fig. 3c) and between insoluble aggregates and misfolding determined by *D*/*N* values (Fig. 3d). Notably high concentrations of detergent-insoluble tau (Fig. 3d, g) and high *D*/*N* ratios (Fig. 3c) in several mice are consistent with CSA curves in these animals; together they indicate extensive inter-individual variability in the levels and conformational characteristics of misfolded tau that is accumulating within congenic TgTau^P301L^ mice.

With samples subdivided by a rostro-caudal cut from sagittal hemibrain samples, approximately twice as much misfolded tau accumulated in the rostral portion of the mouse brains than in the caudal portion (Fig. 3e, f). 75% of mice (*n* = 12) exhibited a higher *D*/*N* ratio in rostral samples, corresponding to patterns of tau deposition detected with AT8 and MC1 antibodies in immunohistochemical analyses [29]. Over a span of 400–800 days, we observed ~ 30% decrease of sarkosyl-soluble tau in older mice (Fig. 3g). This decrease was concomitant with increase in sarkosyl-insoluble tau which peaked at ~ 550 days of age and fit to a Gaussian age-distribution profile (Fig. 3h). Together with an increasing frequency of higher *D*/*N* values, the data indicate age-dependent accumulation of misfolded tau aggregates with a broadening spectrum of conformers (Fig. 3i).

### Tau CSA defines conformational diversity in TgTau^P301L^ mice

**Fig. 4:**
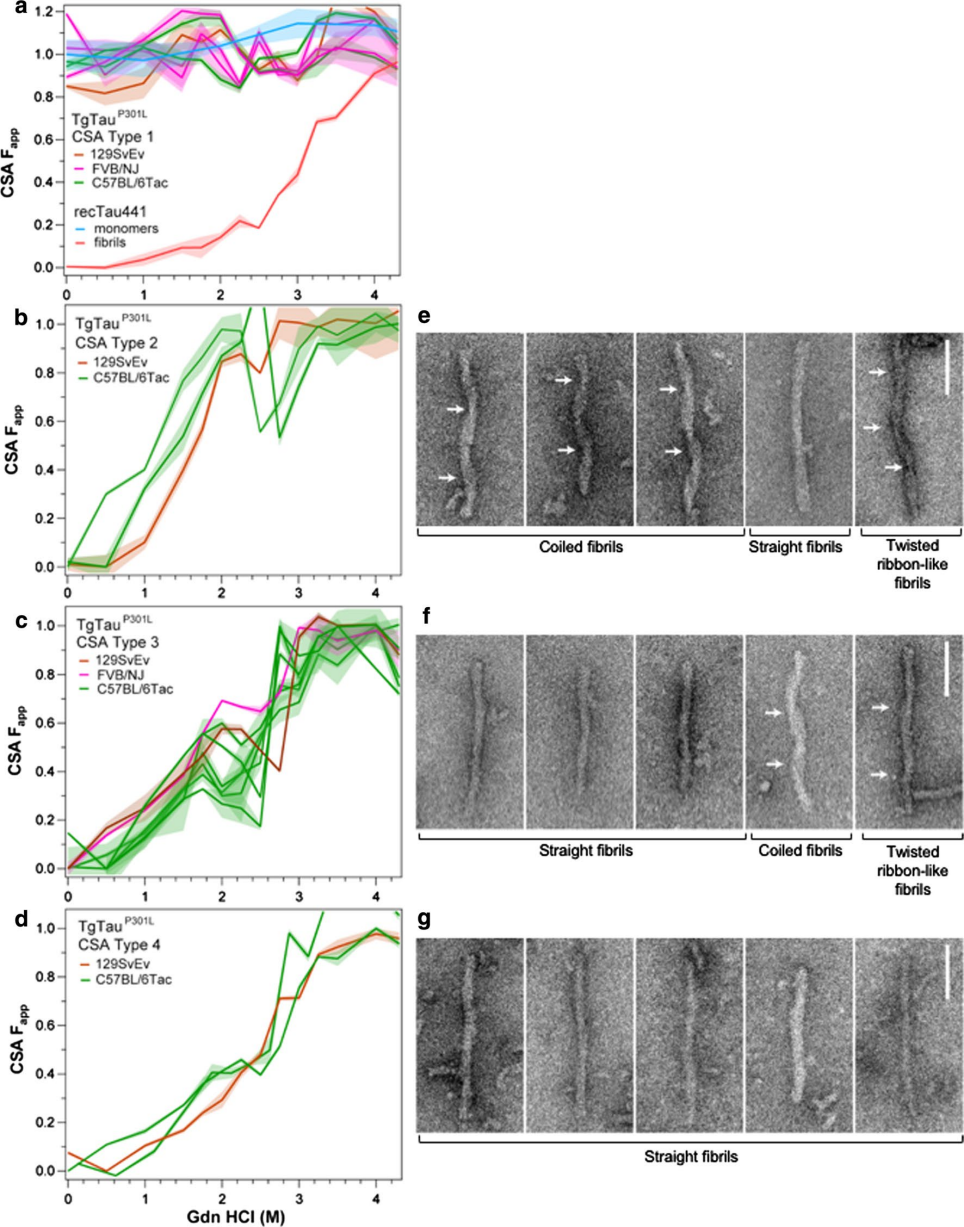
Diverse conformational profiles of sarkosyl-insoluble tau aggregates accumulating in the brains of aging TgTau^P301L^ mice. **a** Mice CSA Type 1 and calibration with monomers and fibrillar aggregates of recombinant tau441 (2N4R); flat signal (turquoise) of monomeric tau441 indicates that both N-terminal epitope (residues 102–139) and R3,4 epitope (residues 316–355) exposed in the native (open conformation [111]) and are buried in the native tau fibrils generating typical one-step sigmoidal transition from native to denatured state (red). **b**, **c**, **d**. Distinct CSA Types 2, 3 and 4 present in TgTau^P301L^ mice; each curve represents dissociation and unfolding in one individual. *F*_app_ values are plotted as mean ± SEM (shades) for each denaturant concentration and assayed in triplicate. Curve analysis was performed with nonlinear regression and significance determined with generalized Wilcoxon test. Average ages (days ± SD) for CSA Types 1, 2, 3 and 4 were 535 ± 32, 649 ± 56, 629 ± 57 and 682 ± 82 days, respectively. Sex distributions were 3–3, 1–2, 7–1, 3–0, female-male for CSA Types 1, 2, 3 and 4. Right-hand panels show corresponding electron micrographs of sarkosyl-insoluble tau protein that was prepared for CSA and CDI assays; for sample sizes, percentage of all morphologies, diameters and periodicities see Table 2 Coiled fibrils predominate in fibrillar tau species seen in a Type 2 CSA sample (44.6%, panels 1–3), with straight fibrils and twisted ribbons less frequent (23% and 12.3%, panels 4 and 5). **f** Straight fibrils predominate in CSA Type 3 samples (87%), with coiled fibrils and twisted ribbons being 1.6% and 1.3% (panels 4 and 5). **g** Type 4 samples almost exclusively contain straight fibrils (96.2%). White arrows indicate cross-overs in coiled fibrils and twisted ribbons. Scale bars = 100 nm

Next, the stability profiles of sarkosyl-insoluble tau species were assessed by titration against incremental concentrations of Gdn HCl denaturant. Recombinant human tau441 in both monomeric and fibrillar states was used as calibrant for development of this CSA. The CSA curve of monomeric recombinant tau441 (which is largely unstructured) was flat, indicating that both the N-terminus and R3, R4 domains are already exposed to antibodies in the native state (Fig. 4a). Fibrillar tau441 generated the expected sigmoidal unfolding curve with a midpoint (c_m_) at ~ 3 M Gdn HCl. Reproducible CSA curves were obtained in 20%, 19% and 40% of aged 129SvEv/Tac, FVB/NJ and C57BL6/Tac TgTau^P301L^ mice, respectively (Fig. 4a–c). The remaining mice had concentrations of sarkosyl-insoluble tau or *D*/*N* ratios (Fig. 3) below the threshold sensitivity of the CSA established with recombinant tau standards. The CSAs from mouse brain samples displayed markedly heterogeneous denaturation profiles of sarkosyl-insoluble tau in individual *T*g mice and by using statistical similarity or dissimilarity of CSA curves in a Wilcoxon test (Fig. 4a–d), we identified four distinct profiles. Mice, defined as CSA Type 1**,** accumulated sarkosyl-insoluble material with only marginal deviation from the recombinant tau monomer curve at ~ 2 M Gdn HCl (Fig. 4a). These animals were euthanized without notable signs of disease and had average ages > 3 months younger than those of animals with CSA Types 2–4 (Type 1 versus Types 2, 3 or 4, *p* < 0.005 in all cases). CSA Type 2 mice displayed a first maximum at ~ 2 M Gdn HCl (Fig. 4b), close to the maximum unfolding at 3 M Gdn HCl. CSA Type 3 profiles showed biphasic transitions with the first maxima at 1.8–2.0 M Gdn HCl (*F*_app_ range from 0.3 to 0.7) and a second transition indicating complete unfolding above 3 M Gdn HCl (Fig. 4c). We have previously described these non-sigmoidal biphasic or triphasic CSA transitions from native to denatured state for human prions and for Aß in AD; they indicate mixtures of different conformers with distinct stabilities [21, 45, 52]. CSA Type 4 profiles were similar to the sigmoidal unfolding curve of fibrillar tau441, but the curves c_m_ shifted by ~ 0.5 M Gdn HCl to a lower concentration, indicating a different conformation with a lower stability (Fig. 4d). The tau concentrations after protease treatment were at the background level indicating that the CSA tau conformers are uniformly protease-sensitive. Consequently, these "below cut-off" levels were insufficient to establish direct CSA profiles of protease-resistant tau.

### Electron microscopy and CSA Types

We also used negative stain electron microscopy (EM) to assess sarkosyl-insoluble material from mouse brain samples versus CSA Types described above (Fig. 4e–g, Table 2). Using re-suspended pellet 3 material, CSA Type 2 profiles with high sensitivity to Gdn HCl were associated with a general paucity of fibrils, but the ones present are represented by coiled, straight and twisted ribbon morphologies (Fig. 4e). CSA Type 4, with a profile similar to recombinant tau, folded into fibrils and with high resistance to Gdn HCl-induced unfolding was found to be exclusively composed of straight filaments lacking a clear helical twist (Fig. 4g). CSA Type 3 profile (Fig. 4f) with a medium sensitivity to Gdn HCl exhibited an intermediate picture with more fibrils than CSA Type 2, but with some coiled fibrils in addition to straight fibril morphology. Last, standing in contrast to fibril morphologies for CSA Types 2–4 (Fig. 4e–g, Table 2), analysis of sarkosyl insoluble material from mice designated as CSA Type 1 yielded a paucity of fibrillar morphologies (Supplementary Fig. 4). Fibril diameters were measured for CSA Types 2–4, along with periodicities for twisted fibrils in CSA Types 2 and 3; diameters of straight fibrils decreased from CSA Types 2–4 (all pairwise comparisons *p* < 0.01) and coiled fibril diameters decreased from CSA Type 2 to Type 3 (*p* < 0.01). In contrast, twisted ribbon-like fibrils increased in diameter from CSA Type 2–3; their associated periodicities of ≥ 130 nm were similar to a reported value for human P301L material [99]. Tau fibrils are known to adopt different quaternary structures in different diseases (reviewed in [40]); our data expand this perspective and correlate structural variations with distinct CSA profiles and stabilities.

### Variations in tau species probed with trypsin digestion and mass spectrometry

Because our CDI and CSA do not differentiate human and mice tau and noting that human 2N4R tau in TgTau^P301L^ animals is expressed at an equal level to net endogenous mouse tau, we used mass spectrometric methods to assess whether protein from the mouse *Mapt* locus is incorporated into sarkosyl-insoluble complexes along with transgene-encoded human P301L protein. This proved to be the case (Supplementary Fig. 5). The mouse peptide HVPGGGSVQIVYKPVDLSK, containing the equivalent of residue 301 at the third amino acid position, was found in all cases examined in a 25–37 kDa size fraction (but not higher molecular weight size fractions) containing the equivalent human peptide HVLGGGSVQIVYKPVDLSK. These data are similar to results obtained with a mouse tau-specific antibody, where co-deposits were observed in rTg4510 0N4R P301L mice [81]. Previous studies have shown that co-expression of endogenous mouse tau does not interfere with propagation and accumulation of pathological forms of human tau but may contribute to neurotoxicity [112]; in our case, since the HVLGGGSVQIVYKPVDLSK mouse peptide was uniformly detected in sarkosyl-insoluble tau from mice with different chemical signatures, its co-existence with human tau may represent a systematic effect.

**Fig. 5:**
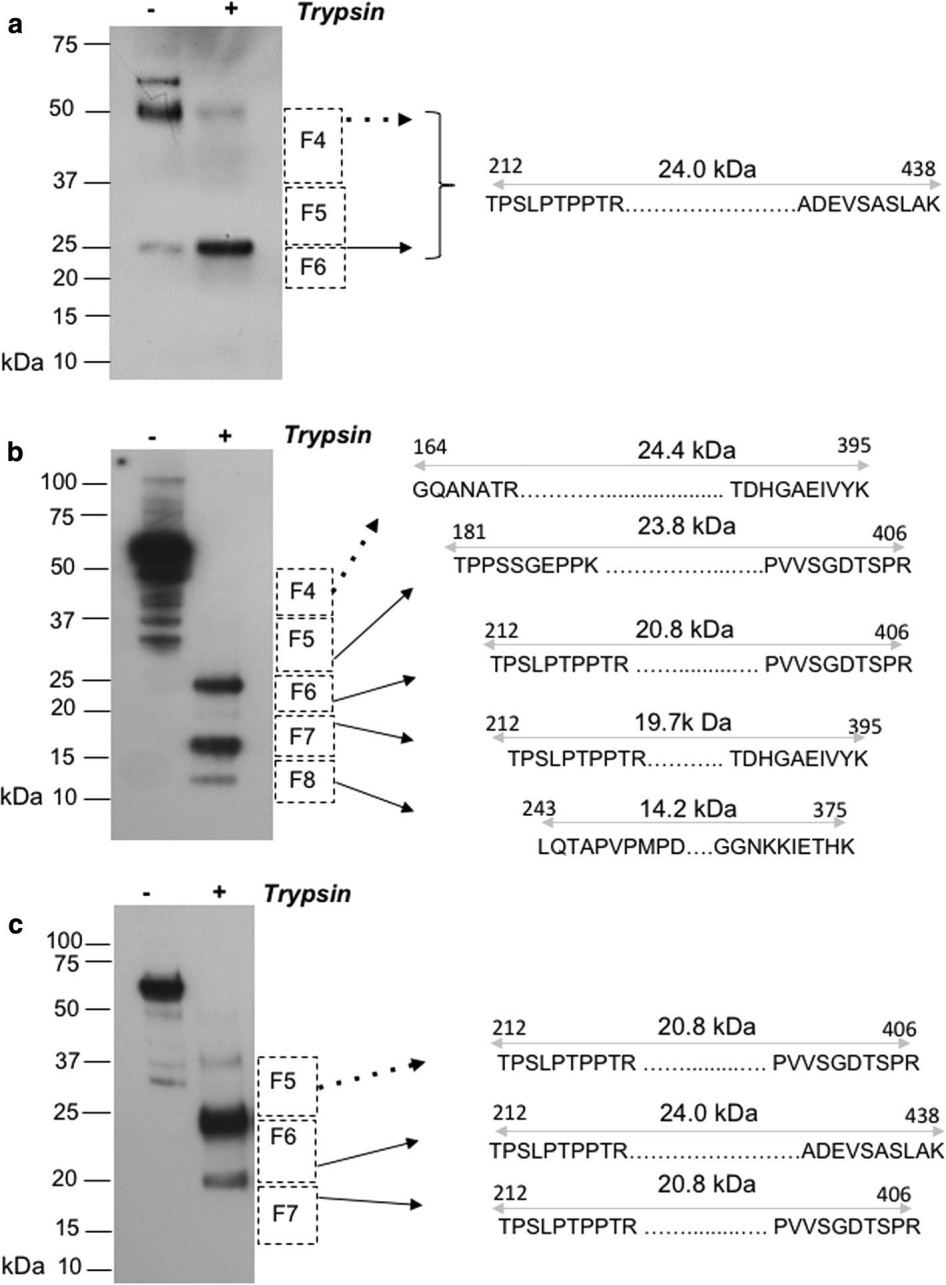
Protein sequence of trypsin-resistant core of TgTau^P301L^ mice identified by LC–MS/MS. The sequences of the peptides identified in different types of trypsin-resistant protein fragment signatures are presented; the boundaries of the trypsin-resistant core fragments are indicated by a sequence of 10 amino acids at the N- and C-termini and by the double-headed gray arrows. Dashed squares adjacent the blots represent size fractions (F4–F8), areas cut from a preparative SDS–polyacrylamide gel and with the black arrows pointing to the corresponding cores found within these size fractions. Arrows with dashed lines indicate cases where the upper limit of the mass spanned by peptides was considerably smaller than the electrophoretic mobility, suggesting the presence of SDS-resistant dimeric forms. **a** Representative blot of trypsin-resistant fragments from mice with a predominantly caudal deposition of abnormal tau detected by IHC (see Supplementary Fig. 6 for epitope-mapping of this core fragment), (**b**) representative blot for a collection of trypsin-resistant fragments found in CSA Type 4 and (**c**) representative blot from a third signature of trypsin-resistant fragments associated with CSA Types 2 and 3. Supplementary Table 1 and Supplementary Fig. 7 present a detailed description of a mouse tau peptide fragment found within the designated size fractions

To bring the trypsin-generated peptides to a detectable level in mass spectrometry (MS), we increased the input of sarkosyl-insoluble material ~ tenfold over that used for PK digestion in CDI experiments. Accordingly, native sarkosyl-insoluble (P3) material was digested with trypsin, fractionated by SDS-PAGE, blotted and probed with the 4R-specific monoclonal antibody ET3 [30]. After in-gel trypsin digestion, liberated internal peptides were detected by liquid chromatography with tandem mass spectrometry (LC–MS/MS). Although mice with caudal tau deposition (Supplementary Table 1) were underrepresented amongst those yielding CSA signatures—5% (1/20) versus 12.2% overall (34/277)—P3 material from the corresponding brain lysates nonetheless yielded a simple trypsin signature of one 25 kDa band and one 50 kDa band (Fig. 5a, Supplementary Table 1). As the 50 kDa trypsin-resistant species did not show a different repertoire of antibody binding than the 25 kDa species (Supplementary Fig. 7), it likely represents a reducing agent and SDS-resistant dimer, as reported previously for a tau protease-resistant repeat core [93]. Detection of the same span of peptides (212–438) in a higher MW fraction from preparative SDS-PAGE gels (fraction 4, 37–50 kDa; Fig. 5a) also supports this interpretation.

CSA Type 4 is at the other end of the spectrum from mouse CSA Type 1 and has a midpoint at a high Gdn HCl value (2.6 M). Multiple trypsin-resistant fragments detected with ET3 antibody in these mice (which uniformly had Class III pathology) shifted to lower MW with corresponding to 25, 18 and 10 kDa signals (Fig. 5b; Supplementary Table 1). In-gel digestion yielded non-phosphorylated peptide signatures spanning approximately 24 kDa (23.8 kDa, residues 181–406 as well as an N-terminally truncated version from 212–406), 19.7 kDa (212–395) and a 14.2-kDa (residues 243–375 as well as an N-terminally truncated version from 282–406) of the complete tau441 open reading frame. The boundaries of the trypsin-resistant 14.2 kDa fragment corresponded closely to the boundaries of the complete microtubule repeat binding region, i.e. residues 243–375 versus 244–368, respectively. Also, a constellation of peptides that indicated a span of 24.4 kDa (residues 164–395) were identified in 37–60 kDa size fractions, again consistent with SDS-resistant dimers. Generally, trypsin-resistant cores in the *T*g mice were more N-terminally extended than those reported for *MAPT* cases (181–406, 212–406 as well as 164–395 and 212–395, versus 260–406, 260–395) [104].

Mice assigned to CSA Types 2 and 3 produced trypsin profiles with an intermediate signature and a trend to longer species (Fig. 5c; Supplementary Fig. 5); while peptide signatures spanning 20.8 and 24.0 kDa (residues 212–406 and 212–438) resembled the CSA Type 4/pathology Class III counterparts, the 12-kDa signature was underrepresented and higher MW species of 37 kDa were apparent by blot analyses. Taken together, the presence of protease and SDS-resistant dimers detected by LC–MS/MS confirm the presence of misfolded tau, each with microtubule binding repeats protected from trypsin digestion. The extent to which different peptide fragment signatures and yields are due to the conformational diversity, differential lysine acetylation**,** or both, will have to be investigated with high-resolution conformationally sensitive MS tools such as hydroxyl radical footprinting and hydrogen/deuterium exchange [61].

### Seeding activity from aged TgTau^P301L^ mouse brains measured in a cellular assay

Given ensembles of tau conformers indicated by physicochemical signatures, we sought other assays that might also support this phenomenon. Samples were next tested in a seeding assay featuring a substrate for tau misfolding comprised of a central region of tau (with P301L and V337M missense mutations) fused to a YFP reporter [94]. Correspondingly, the clone 1 HEK sub-line expressing this construct was exposed to brain homogenates from *T*g mice. Considering the possibility of prion-like conformer selection and evolution [60, 70] in HEK cells due to the V337M mutation, an earlier assay format with an endpoint 30 days after transduction [29] was revised to an observation period of 6 days, to better detect early events and/or isolates that might be under a selective disadvantage in a longer observation period. Besides testing sarkosyl-insoluble material, "P3", fractions from an earlier step in a sequential purification protocol was also assessed (see Materials and Methods, [29, 92, 104]). Noting CSA signatures may be driven by oligomeric species, we also tested a soluble fraction, supernatant 1 (“S1”) in the seeding assay.

**Fig. 6:**
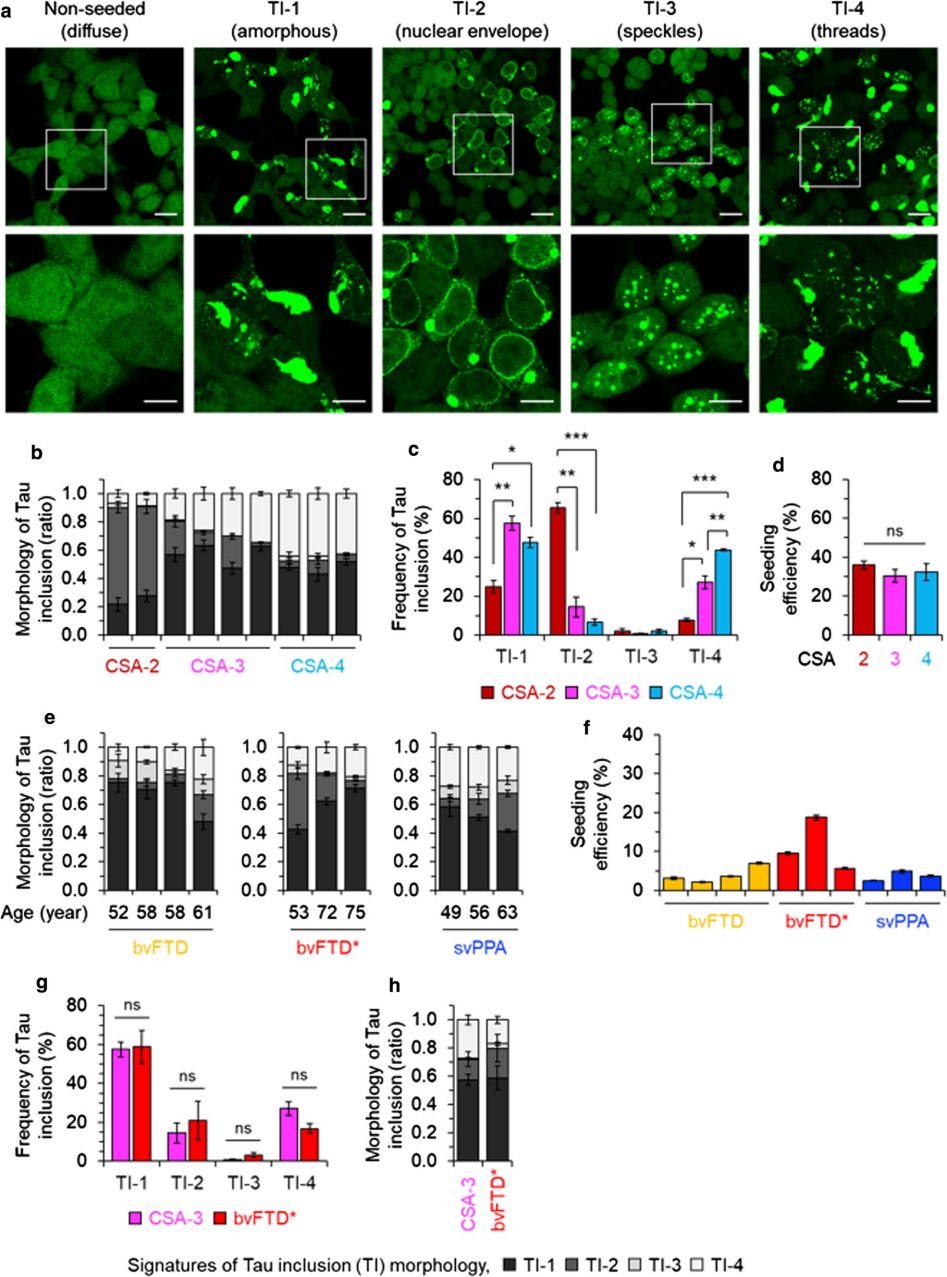
Strain-sensitive differential tau seeding assay using YFP reporter cells. **a** HEK-tauRD-LM-YFP reporter cells express YFP-fused to a human tau repeat domain (RD) with P301L and V337M mutations. In the non-seeded control cells YFP signals are not focal. Cells seeded with tau protein revealed various morphologies of tau inclusions (TIs) characterized by differences in their subcellular distribution; a large mass of aggregated tau (amorphous: TI-1), nuclear envelope and juxtanuclear inclusions (nuclear envelope: TI-2) (reminiscent of “mini-Pick-like bodies'' in granule cells of the dentate gyrus of human cases), granular nuclear inclusions (speckles: TI-3) and thread-shaped inclusions (threads: TI-4) (also seen in human cases). Scale bar, 20 µm and 10 µm in the boxed images. **b**–**d** data from mouse brains. Ratio (**b**) and frequency (**c**) of TIs observed in HEK-tauRD-LM-YFP reporter cells after seeding with TgTau^P301L^ mouse brains, grouped by Type of CSA signature (types 2, 3 and 4) and normalized by total concentration of tau. Error bars represent SEM. **p < 0.01 and ***p < 0.001. ns, not significant. **d** Relative seeding efficiency of TgTau^P301L^ brains of the different CSA Types analyzed in panels (**a**–**c**). Samples were normalized by tau amounts determined by immunoassay. **e**, **f** Analyses for human frontal cortex samples (S1 fraction) assigned different diagnoses (Table 1). **e** Data plotted as per panel (**b**) also including age at death for individual cases. **f** Seeding efficiency versus clinical diagnosis; higher efficiencies in bvFTD* samples did not reach significance. **g**, **h** Averages of individual mouse brain samples with a CSA Type 3 profile showed similarities to averaged human bvFTD* samples when assessed in pairwise comparisons for inclusion types (**g**) or plotted by ratios of inclusion morphologies (**h**)

These protein transduction experiments defined multiple fluorescent morphologies associated with the material from single mouse brains, irrespective of whether the starting material was “P3” or “S1” (Fig. 6, Supplementary Fig. 6). The experiments revealed four signatures of tau inclusions (TIs, “TI-1 to 4”, Fig. 6a**)**, three being similar to morphologies described by Sanders and co-workers [94] and with TI-2 being dissimilar. Analyzing S1 samples from different mouse brains revealed different proportions of the different types of inclusions. TI-1 resembled the signature seen in experiments with a longer observation period [29], but other fluorescent signatures were also present; these comprised decoration of the nuclear membrane with an adjacent focal cytoplasmic signal (TI-2), speckles (TI-3) and cytoplasmic threads (TI-4). It was evident that the ratios of TI-1 to 4 signals differed with the CSA Type of the donor mouse brains (Fig. 6b). CSA Type 2 differed significantly from Types 3 and 4 for the abundance of TI-1, -2 and -4 fluorescent signatures (*p* < 0.01, 0.001, Fig. 6c) and all three CSA Types were significantly different for TI-4 signatures (*p* < 0.01, 0.001, Fig. 6c). Individual transduction efficiencies for mouse CSA Types 2–4 were not distinguishable (Fig. 6d). Some similarities between the organelle distribution of fluorescent signals scored in the reporter cells and histopathological findings in brain tissue are discussed below. Regarding the use of human FTLD-MAPT-P301L brain material, while protein transduction frequencies were on average approximately 1/17 lower than for mouse samples, perhaps reflecting a reported effect of age [1], we scored the same types of inclusion morphologies TI-1 to 4 (Fig. 6e, f). Samples from different classifications made at the time of presentation gave different proportions of these signatures (Fig. 6e). In terms of the frequency (percentage) of different fluorescent inclusion morphologies, CSA Type 3 from mice and FTLD-MAPT-P301L cases denoted as bvFTD* were not significantly different (Fig. 6g, h; Table 1).

### Tau conformer strains in Tg mice versus FTLD-MAPT-P301L cases

**Fig. 7:**
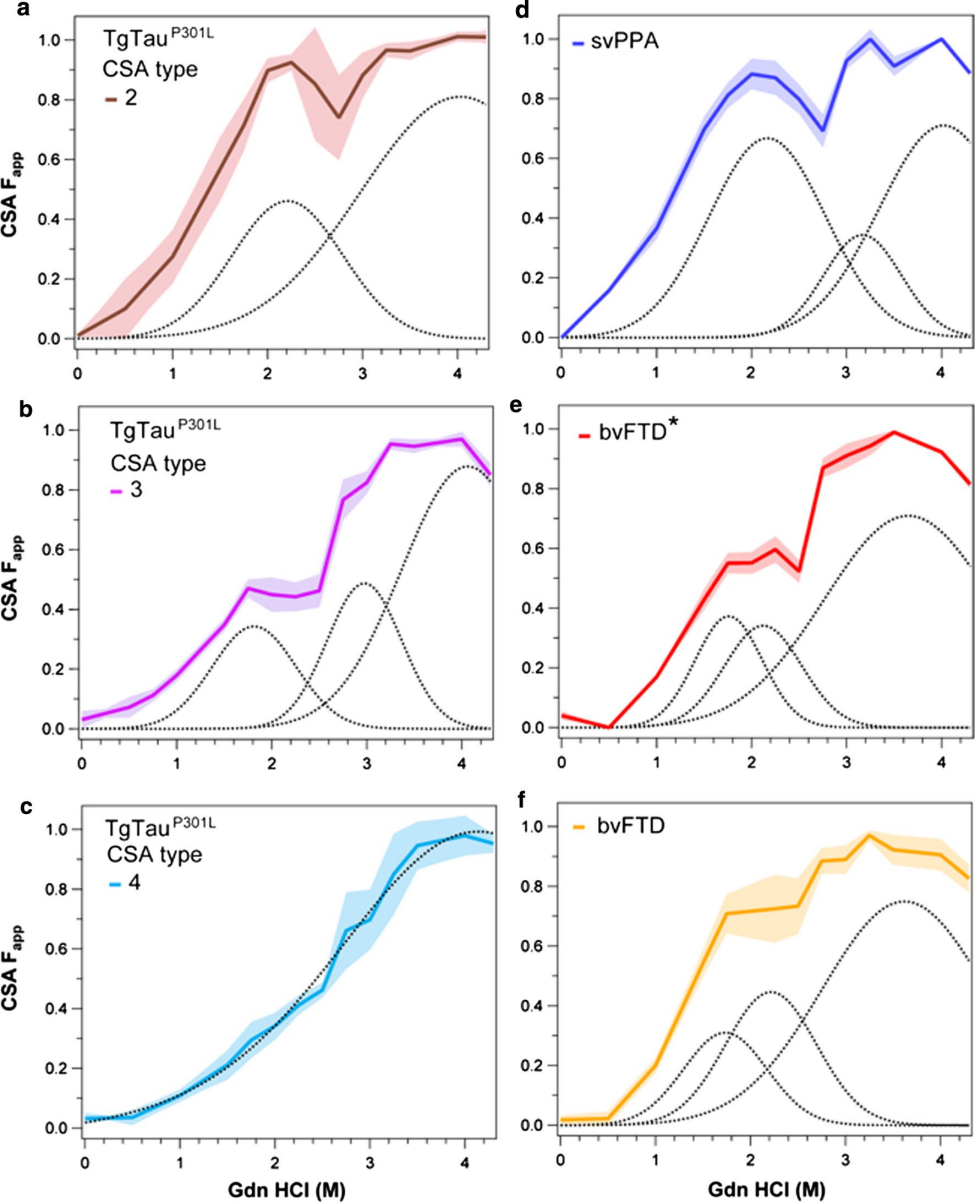
Comparison of conformational profiles of sarkosyl-insoluble tau averaged in different subtypes of TgTau^P301L^ mice and in FTLD-MAPT-P301L cases. **a**, **b**, **c** Averaged profiles and statistical mechanical Gaussian peak deconvolution of sarkosyl-insoluble tau profiles in Type 2 (**a**), Type 3 (**b**) and Type 4 (**c**) CSA subtypes in TgTau^P301L^ mice. (**d**, **e**, **f**) Averaged CSA profile and Gaussian peak deconvolution of CSA profiles of detergent-insoluble tau in cases with the following clinical diagnoses: semantic variant of Primary Progressive Aphasia (svPPA), behavioral (bvFTD) and memory variant of bvFTD (bvFTD*). Curve deconvolution was performed with a multiple peak Gaussian model and the statistical significance was determined with generalized Wilcoxon tests

With the exception of CSA Type 4 profile in TgTau^P301L^ mice (Fig. 4d), all other tau CSA curves were non-sigmoidal, indicating more than a single transition from native to denatured state. We analyzed these profiles using statistical mechanical deconvolution and Gaussian models previously applied to proteins that undergo multi-step thermal denaturation [3, 91]. Apart from mouse CSA Type 4, Gaussian deconvolution of averaged profiles in samples with non-sigmoidal tau denaturation profiles revealed two or three peaks (Fig. 7a, b versus 7c**)**, each representing conformers with different stabilities that are unfolded sequentially with Gdn HCl. These data provide evidence that each tau CSA subtype is a mixture of up to three distinct conformers. Of four tau CSA signatures analyzed in TgTau^P301L^ mice, Types 2 and 3 (Fig. 7a, b) showed similarity with human FTLD-MAPT-P301L tau profiles before protease treatment (Fig. 2d), with prominent conformers unfolded at ~ 2 M Gdn HCl; mouse Type 2 was a statistical match with svPPA profile (Fig. 7d; *P* = 0.2673, two-tail Wilcoxon). Comparing amongst the human P301L cases, the averaged svPPA profile was significantly different from both bvFTD (*P* = 0.009) and cases scored as bvFTD* (*p* = 0.006) (Fig. 7d–f). While bvFTD and bvFTD* cases were not distinct from each other (*p* = 0.2460), these analyses of protease-sensitive conformers indicate at least two strains of tau strains in FTD-MAPT P301L brains (noting that all three phenotypic variants of FTD showed similar profiles after PK treatment; Fig. 2e). A summary of statistical comparisons of the human cases is presented in Supplementary Table 3. Neuropathological examination using AT8 antibody did not reveal obvious differences in the amount, distribution or morphology of AT8 immunoreactive structures among the different clinical phenotypes (Table 1).

While it is possible that further subtle conformational changes await discovery (perhaps requiring more sensitive biophysical and pattern recognition approaches to achieve statistical significance), these overall data provide evidence for extensive conformational heterogeneity of misfolded tau P301L, with three subtypes of tau conformer profiles (i.e. CSA Types 2, 3 and 4) evolving spontaneously in aged TgTau^P301L^ mice and three profiles of tau isolates in FTLD-MAPT-P301L cases. The remaining mouse CSA profile, Type 1, occurred in mice slightly younger than in Types 2–4 and is interpreted to represent an early step in pathogenesis (and with mice failing to reach CSA criterion likely representing a yet earlier step). The Type 2 and 3 conformer profiles from TgTau^P301L^ mice are similar to the profiles of svPPA and bvFTD/bvFTD* profiles, respectively. Of note, echoing the similarities between the average mouse CSA Type 3 profile and the average profile of cases diagnosed as bvFTD* cases (Fig. 7b, e), the averaged seeding repertoires of these types of mouse and human samples were not significantly different (Fig. 6g, h). While mouse CSA Type 2 profiles resembled those of svPPA samples (Fig. 7a, d), this was not so for the cellular assay, perhaps reflecting a seeding barrier effect due to the extra M337V mutation in the YFP-tau reporter construct.

**Fig. 8:**
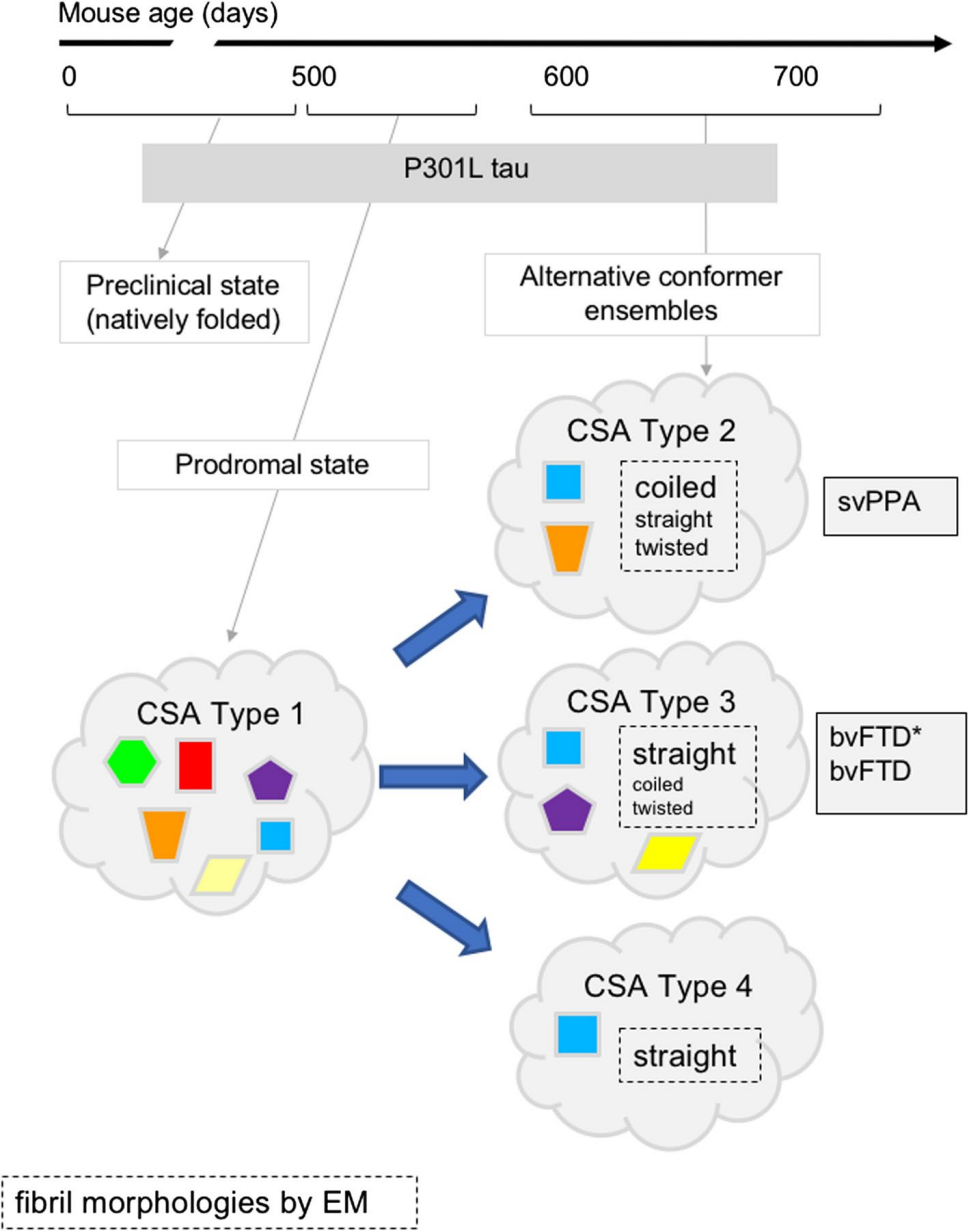
Evolving conformer ensembles in the pathogenesis of a primary tauopathy. Conformers of protease-sensitive detergent-insoluble tau in TgTau^P301L^ mice are represented by different geometric shapes. Different coexisting combinations (i.e., ensembles) of conformers corresponding to different CSA profiles are shown within the cloud outlines [24]. In some cases, the types of fibrillar assemblies associated with the ensembles are also presented, with a larger font indicating the most abundant species (boxes with dashed outlines). CSAs Types 2–4 are seen in mice with indistinguishable average ages (Fig. 4 legend) and hence likely represent alternative pathways of ensemble evolution (blue arrows). The closest equivalent human disease profiles to mouse CSA Types 2 and 3 are also presented (boxes with solid outlines)

In sum, even within an extensive heterogeneity of tau isolates in FTLD-MAPT-P301L cases, the distinct conformational characteristics of protease-sensitive tau species (but not protease-resistant tau aggregates) were associated with different clinical phenotypes. Moreover, and importantly, with the exception of CSA Type 4 in TgTau^P301L^ mice, the other tau signatures are not indicative of a single conformational entity, but denote a mixture of at least two or three distinct conformers, i.e. a strain mixture (summarized in Fig. 8).

## Discussion

A prior seminal observation of different patterns of tau aggregates cloned in HEK cells from patients with distinct tauopathies has been made by Diamond and co-workers [95], with more recent studies suggesting that different tau strains can be generated from monomers without seeds [64, 98]; the latter data have been interpreted as evidence that alternatively structured monomers can encipher the information essential for formation of tau strains. As our analyses did not include techniques to single out monomeric species, we cannot speak directly to this concept. However, our data are compatible with a stepwise process for the emergence of tau strains. In the earliest events we characterized in our low-expresser model that P301L tau generates a cloud (spectrum) of insoluble tau aggregates with different structural organizations; these can then evolve down alternative pathways to generate conformationally distinct tau strains. This is compatible with data showing that increased conformational flexibility due to the P ≥ L substitution does not significantly increase beta-structure in monomers, but instead promotes acquisition of beta-structure driven by intermolecular interactions and aggregation [31]. Further supporting this interpretation, each tau isolate—here defined operationally by deriving from an individual mouse brain or a dissected brain region from an individual FTLD-MAPT-P301L case—is rarely a single conformational entity but typically a mixture of up to three different conformers that together may give rise to distinct neurological phenotypes.

In the case of sporadic prion disease, the explanations tendered to account for this etiology include spontaneous somatic mutations in the prion gene *PRNP* or rare stochastic conformational changes to create misfolded proteins [77, 86]. Whether age-related tauopathies are the result of such primordial events remains to be established, but our data imply that from a first triggering step of a germline *MAPT* mutation, misfolded tau proteins emerge from a nascent ensemble of sarkosyl-insoluble species into different spectra of strains (Fig. 8). For misfolded PrP, the concept of natural selection and evolution (e.g., acquisition of drug resistance) has been applied for human and rodent prions, which undergo progressive conformational shifts due to the natural selection of conformers with the highest replication rate [45, 70]. The same mechanisms of conformational selection likely apply to misfolded tau aggregates and our data suggest a major role of protease-sensitive oligomers. However, investigating this Darwinian selection process at a conformational level requires high-fidelity cellular and in vitro propagation methods, which remain to be developed for tau strains [52, 60]. Moreover, in contrast to a prion replication process that is predominantly extracellular, tau is predominantly intracellular with its own repertoire of cell biological phenomena wherein the *MAPT* gene—transcribed perhaps exclusively in neurons [41, 55] and driven in the *T*g mice by a neuronal promoter from the hamster PrP gene [96]—can nonetheless be deposited in astrocytes and oligodendrocytes [29, 69]. It seems likely that the associated cell biological events in these lineages, including response to excitation and proteostatic repertoire will have their own selection pressures that sculpt repertoires of tau conformers [35]. Intriguingly, the data presented here begin to hint at cellular organelles and compartments that may prove relevant to these selection processes. Thus, association with nuclear membrane illustrated by TI-2 fluorescent signals (Fig. 6a) brings to mind reports of tau within the nucleus and in perturbations of the nuclear membrane and import/export processes [9, 15, 28, 73, 102]. These types of tau inclusions may warrant closer examination in the FTLD-MAPT-P301L cases to seek analogous structures. Also, TI-1 and TI-2 signatures with compact fluorescent signals in the cytoplasm bear some resemblance to “mini-Pick-like bodies” reported in dentate gyrus neurons of the P301L cases used here [7].

Our observations call for a re-evaluation of therapeutic strategies targeting protein isoforms that are already misfolded and a need for high-resolution structural tools to study the impacts of protein ligands and posttranslational modifications on conformational transitions. Considering pathogenic conformers as potential targets, chemical signatures indicate that these conformers are rarely seen in pure form ‘in isolation’ but are typically found as ensembles of species (Fig. 7). A partial analogy may be found in the observation that more than 30% of sporadic CJD cases involve 2 human prion strains in the same or different brain locations [16, 74, 79, 107] and this type of strain coexistence effect may be heightened in tauopathies; indeed, the view that singular tau strains drive singular clinical entities may need revisiting. Furthermore, there are firm indications that conformer ensembles are neither unique nor static; they may emerge from a cloud of species in a prodromal state and then diverge under selection pressure in a sub-clinical phase towards the alternative ensembles and corresponding signatures scored in the clinical phase (Figs. 2, 3, 4, 5, 6, 7, 8). Correspondingly, therapies will need to address the molecular mechanisms responsible for the natural selection process in different cell populations. In parallel, there will be a need to address the substrate for misfolding, i.e. natively disordered tau. In prion disease, targeting the native precursor rather than misfolded products has the potential to circumvent PrP^Sc^ “strain evasion” effects [11, 60]. This approach is based upon the absence of an overt phenotype in homozygous null animals lacking PrP^C^ [5, 14, 25, 82] and almost threefold extended lifespan of prion-infected mice after downregulation of PrP^C^ [87]). Tau knock-outs in mice are known to be viable [49], suggesting that knock-down of tau mRNA might be a straightforward approach to block the formation and diversification of toxic tau conformers.

## Electronic supplementary material

Below is the link to the electronic supplementary material.

Supplementary file1 (PDF 7099 kb)
