# Supplementary material for: Diverse, evolving conformer populations drive distinct phenotypes in frontotemporal lobar degeneration caused by the same MAPT-P301L mutation
Source: Acta Neuropathol. 2020 Mar 26;139(6):1045–70. doi: 10.1007/s00401-020-02148-4 (PMC7244472; doi:10.1007/s00401-020-02148-4)
Supplement: Supplementary file 1 — Supplementary file1 (PDF 7099 kb) [file 401_2020_2148_MOESM1_ESM.pdf]

SUPPLEMENTARY DATA

Supplementary Figures.

Supplementary Figure 1. Seeking integrated reverse transcripts in mouse brain genomic DNA. (a) Position of the PCR primer pair (black arrows) on either side of a hamster PrP gene intron in the transgene vector; their distance apart (grey arrowheads, kilobase pairs) precluding PCR amplification from gDNA with the chosen polymerase extension time. Lower panel shows the 0.62 Kb PCR product if extraneous cDNA copies of the tau transgene are integrated in other loci (dashed line). **(b)** Agarose gel of nested PCR from gDNA extracted from $TgTau_{P301L}$ brain homogenates; gDNA was 100 ng per PCR reaction, 33,000 genome equivalents. Mice were classed as per **Supplementary Table 1**. The expected size of the amplification product from a reverse transcript of the transgene (i.e. lacking the intron) is marked with an open arrowhead. No individuals are positive for expected fragment. Smaller amplification products were sequenced and were unrelated to *MAPT* sequences. “n” = negative control with no genomic DNA template. Size markers were 1500, 1000, 700, 500, 400, 300, 200 and 75 base-pairs (bp). **(c)** Agarose gel of nested PCR performed as above, with a positive control 3608 bp template from $2.57x10_6$ to 2.57 copies added to the primary PCR along with mouse gDNA. Approximately 26 copies of the gene are sufficient to amplify the intended target in a reaction that contains 33,000 genome equivalents, hence a detection limit of one part in 1270. The 624 bp PCR amplicon is designated as in **(a)**.

Supplementary Figure 2. Broad linearity range of tau441 measurement using two

different CDI formats. These formats were originally developed for detection and differentiation of mammalian prions [85,61,52]. **(a)** Sandwich CDI using plates coated with DA9 capture antibody. **(b)** Direct format with tau protein adsorbed onto the high-binding plastic surface of the ELISA plates.

Supplementary Figure 3. Normal levels of Aß 42 with D/N ratio ~1 in FTLD-MAPT-P301L cases indicate low concentrations of Aß monomers and no pathogenic aggregates. The CDI for Aß 42 was performed as described and compared with published data [23]. “F” and “C” represent frontal cortex and cerebellar samples, respectively, from the FTLD-MAPT-P301L cases. The y-axis represents concentration of Aß 42 in ng/ml and the Aß 42 D/N ratio outlined on the x-axis (**Table 1**).

Supplementary Figure 4. Heterogeneous aggregates seen in CSA Type 1 mouse brain samples.

Left panel: Amorphous aggregates seen in sarkosyl insoluble material from mouse brain samples defined as having a CSA Type 1 profile. These samples also contain occasional fibrillar tau species, but these were in the minority. Middle panel: Sarkosyl insoluble material from a CSA Type 2 sample showing a majority of characteristic tau fibrils (compare **Fig. 4f**). Right panel: Equivalent view from a CSA Type 3 sample, again showing predominantly tau fibrils (compare **Fig. 4g**). Scale bars = 200 nm.

Supplementary Figure 5. MS/MS spectra show a mixture of WT and mutant P301L fragments in the trypsin resistant core. a. Spectrum of peptide H299VPGGGSVQIVYKPVDLSK317 from the WT Tau sequence. [Charge: +3,

Monoisotopic m/z: 660.70276 Da (+0.78 mmu/+1.19 ppm), MH+: 1980.09372 Da, RT: 25.61 min]. **b.** Spectrum of the peptide H299VLGGGSVQIVYKPVDLSK from the mutant sequence (P301L). [Charge: +3, Monoisotopic m/z: 666.04620 Da, MH+: 1996.12406 Da, RT: 39.23 min]. Ions observed are indicated in the peptide sequence. The charge of the ion series identified is included (superscript). The mass tolerance for the fragment ions was set to 0.01 Da, charged ions +1 to +4 and modifications b, b-H_2O, b-NH_3, y, y-H_2O and y-NH_3 were considered.

Supplementary Figure 6. Cell seeding activity of P3 and S1 fractions. (a) HEK-tauRD-LM-YFP reporter cells were seeded with P3 and S1 fraction of a Class II $TgTau_{P301L}$ mouse brain and imaged at 6 days after seeding. Scale bar, 40 µm and 20 µm in the boxed images. **(b, c)** Ratio **(b)** and frequency **(c)** of TIs induced by two mouse brains showing different types of tau deposition defined by immunostaining with AT8 antibody (Class II or Class III; see Table S1) [29]. Error bars represent SEM. *$p < 0.05$. **(d)** Seeding efficiency of P3 and S1 fractions of each brain.

Supplementary Figure 7. Epitope mapping of trypsin-resistant tau species. Epitope mapping of the trypsin-resistant cores in samples as per Fig. 5a. **(a)** Two brain S3 samples were incubated plus or minus trypsin digestion and independent gels were blotted with CP27, TG5 and ET3 anti-tau antibodies with epitope positions as shown; absence of the 50 kDa band in CP27 and TG5 blots suggests this protein species is an SDS-resistant dimeric form of the 25 kDa fragment. **(b)** Spiking experiment to demonstrate lack of cross-reactivity between ET3 antibodies and recombinant trypsin used to digest P3 samples. S1 designates undigested supernatant 1 of a 457-day-old

$TgTau_{P301L}$ mouse brain. Digest buffer contained (l to r) 0, 0.5 and 10 mg of trypsin. The open arrow designates the anticipated position for a cross-reactive signal.

Supplementary Fig. 1

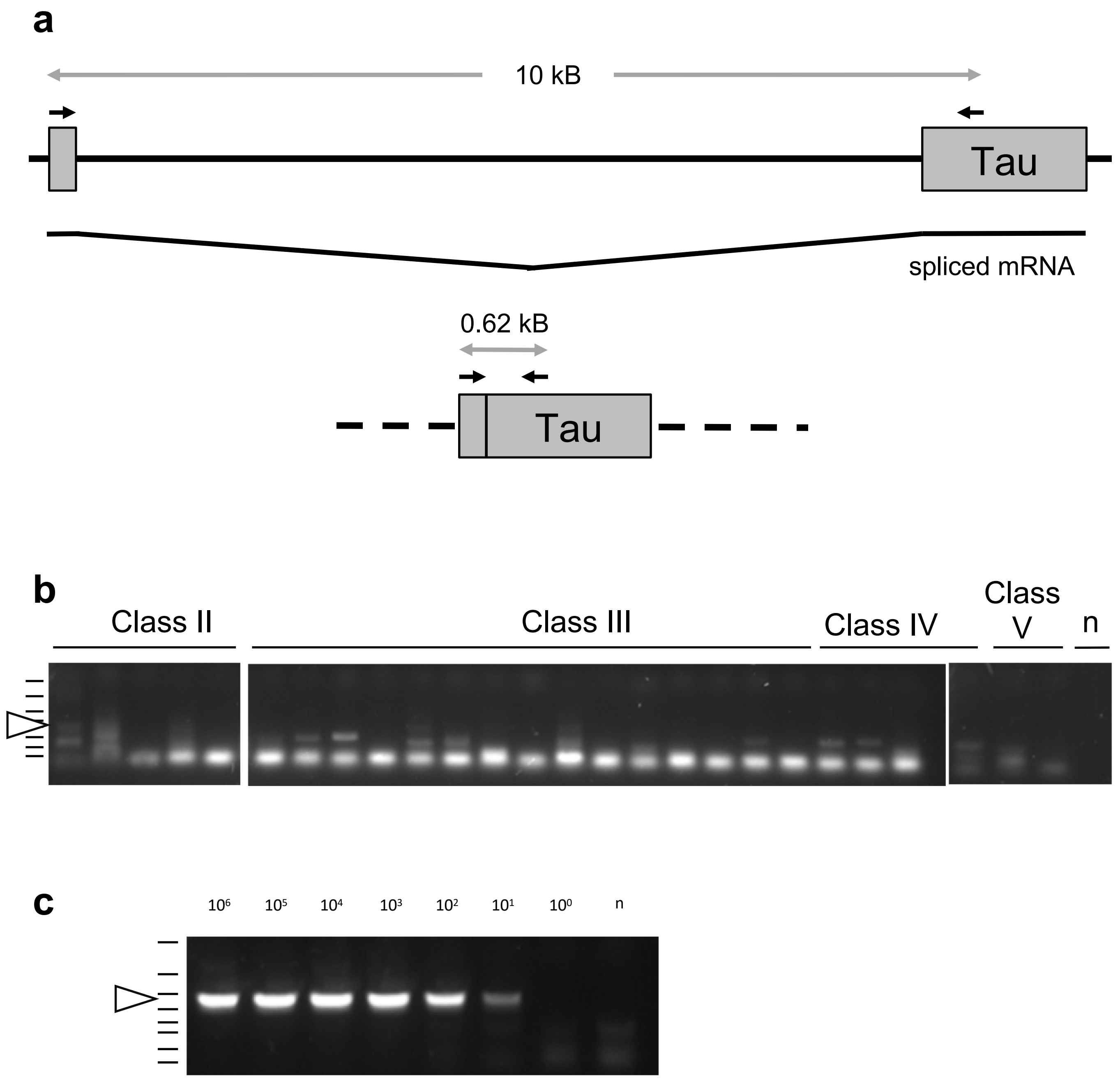

Supplementary Fig. 2

a

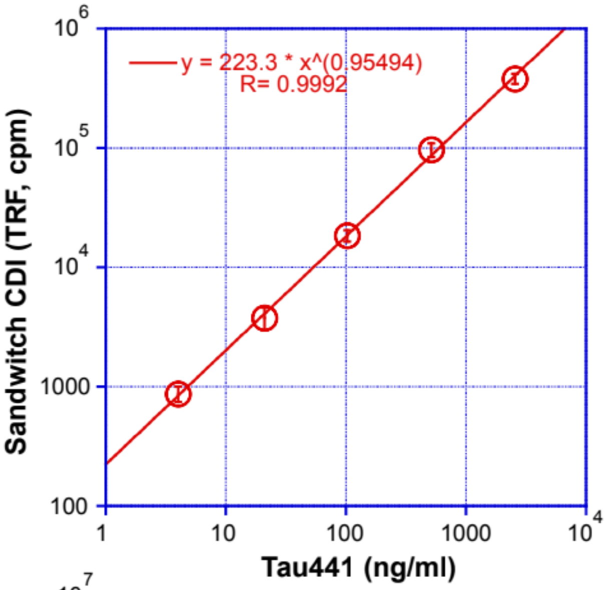

b

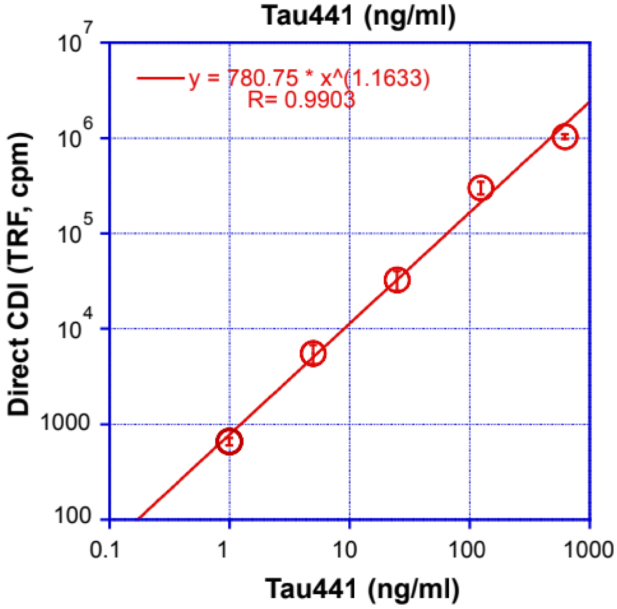

Supplementary Fig. 3

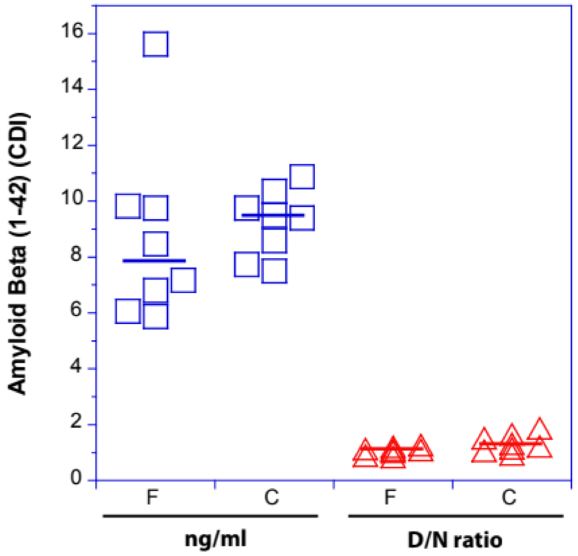

Supplementary Fig. 4

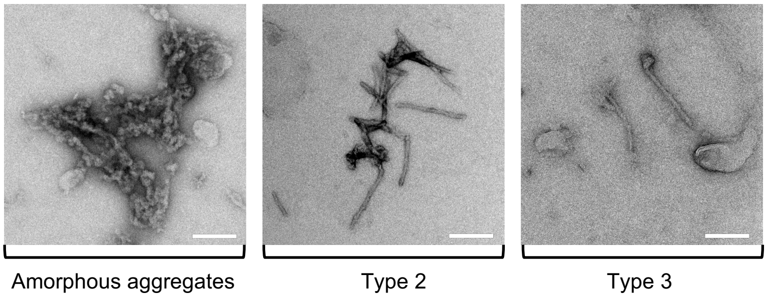

Supplementary Fig. 5

a

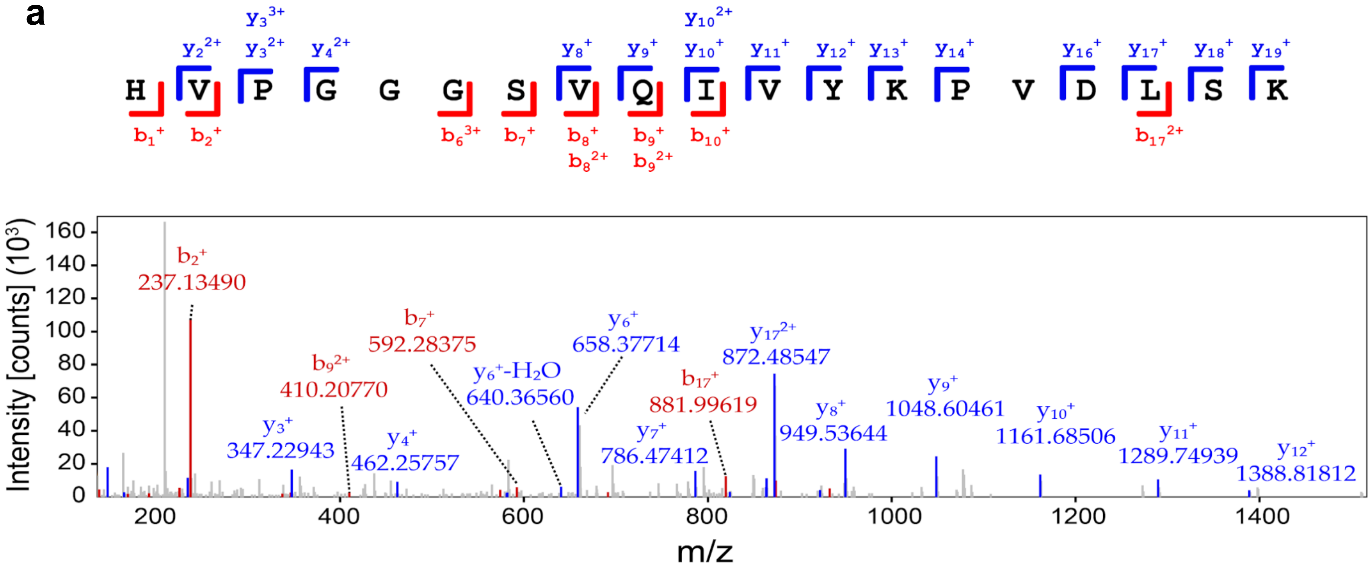

b

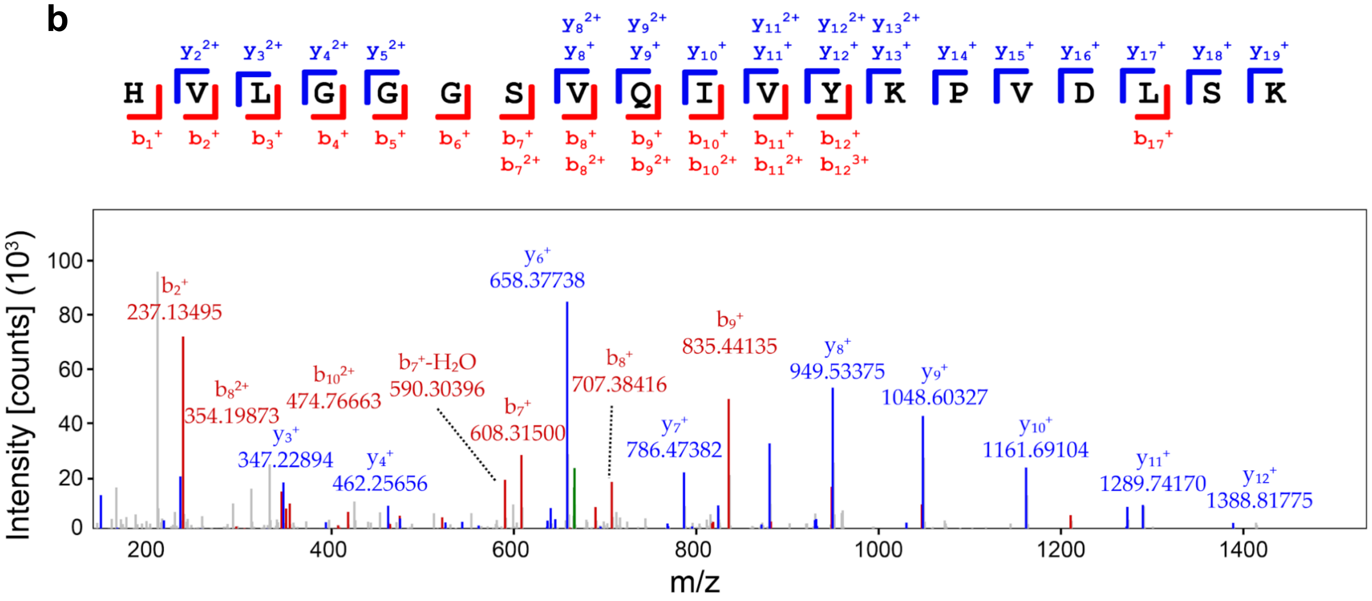

Supplementary Fig. 6

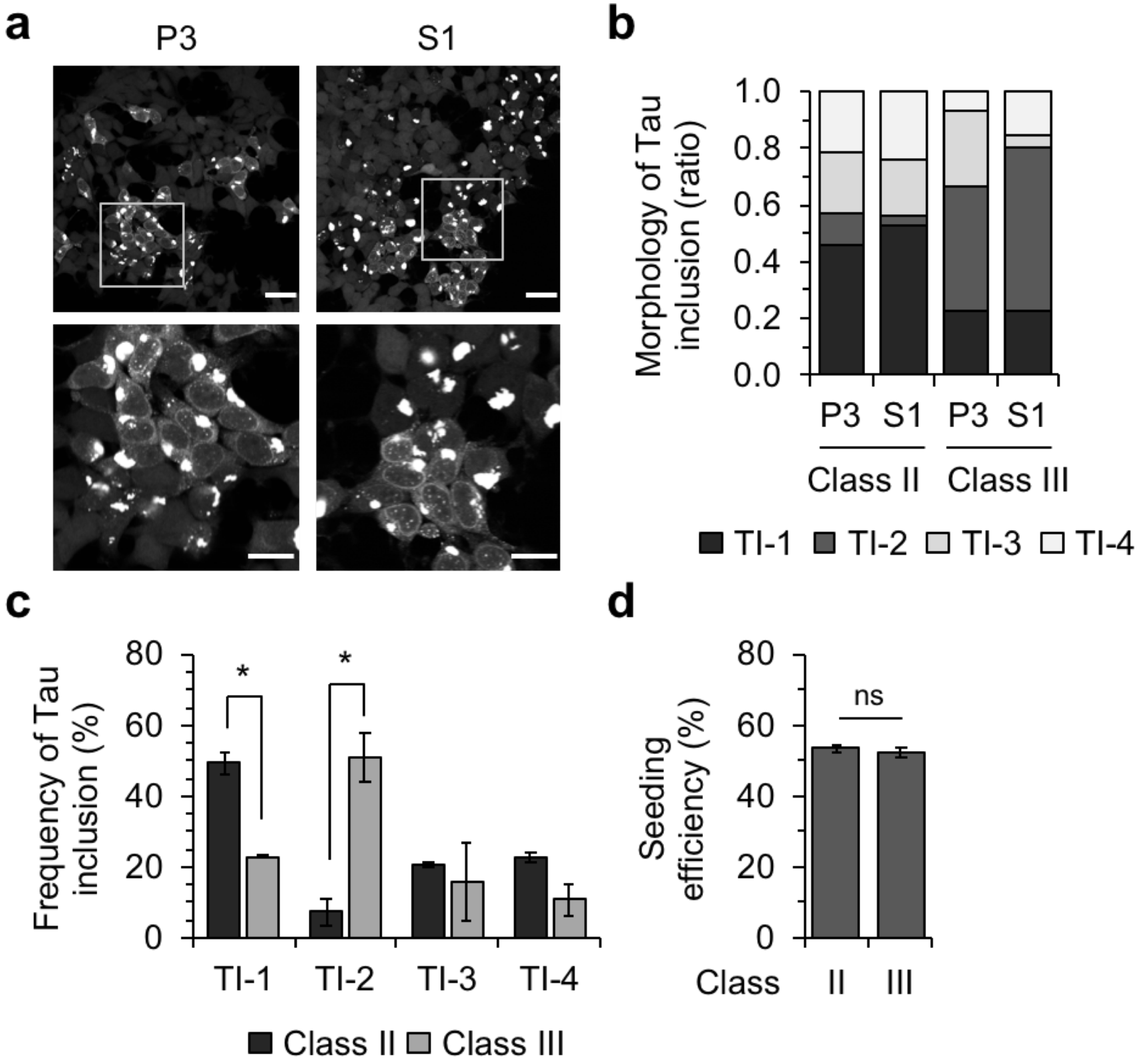

Supplementary Fig. 7

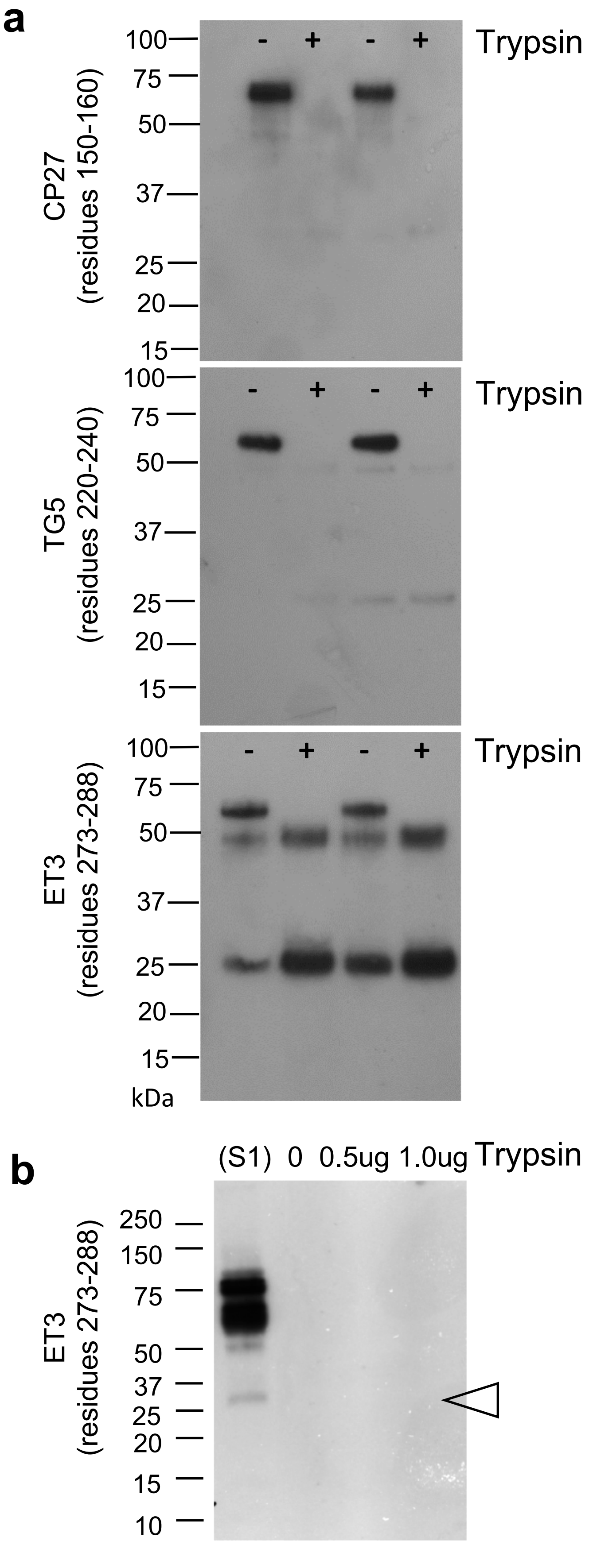

Supplementary Table 1. Age distribution and pathology classes in $TgTau^{P301L}$ mice with different genetic backgrounds.

Pathology Class, age in days ± SD (n♀/♂)	Genetic background (n ♀/♂)			Predominant pattern of trypsin-resistant core (n/N)
	**C57BL/6Tac (44/35)**	**129/SvEvTac (48/53)**	**FVB/NJ (34/29)**	
I	603 ± 96 (5/6)	595 ± 77 (3/2)	526 ± 65 (5/13)	A (3/6)*
II	612 ± 65 (9/9)	600 ± 84 (8/13)	571 ± 73 (5/5)	A (7/8)
III	611 ± 70 (24/10)	628 ± 81 (25/25)	601 ± 88 (12/6)	B (6/7)
IV	526 ± 42 (4/7)	546 ± 38 (7/4)	513 ± 72 (9/3)	C (4/4)
V	609 ± 55 (2/3)	597 ± 72 (5/9)	510 ± 56 (3/2)	C (2/2)

Modified from [23]. The inbred strain distribution of mice trypsin resistant core patterns A, B or C was 7, 13, 11 (n=31) for C57BL/6Tac, 129/SvEvTac and FVB/NJ, respectively. One sample each from classes I, II and III had no detectable signal while 4 animals with unclassified patterns of focal tau deposition had trypsin-resistant signature of pattern C. * 2/9 mice had trypsin-resistant signature of pattern C.

Supplementary Table 2. Examination of FTLD-MAPT-P301L cases for co-existing proteinopathies.

				Other staining in the dentate gyrus				
**Case**	**Diagnosis**	**Age**	**Sex**	**Tau**	**BA4**	**Asyn**	**TDP43**	**FUS**
**14**	Control	70	M	0	0	N/A	N/A	N/A
**17**	Control	81	F	0	2+ (a)	0	N/A	N/A
**22**	Control	31	M	0	0	0	0	N/A
**25**	Control	78	M	1+ (b)	0	0	0	N/A
**26**	Control	76	M	1+ (c)	0	0	0	N/A
**27**	AD	92	F	2+ (d)	2+	0	1+	N/A
**18**	AD	72	F	1+ (e)	2+	0	N/A	N/A
**15**	GRN	60	M	0	0	0	2+ (f)	N/A
**23**	GRN	64	M	1+ (b, f)	0	0	2+	N/A
**16**	ALS	50	M	0	0	0	0	N/A
**19**	ALS	64	M	0	0	0	0	N/A
**21**	ALS	51	M	0	0	0	0	N/A
**24**	ALS	63	M	0	0	0	0	N/A
**28**	ALS	50	M	0	0	0	0	N/A
**20**	FTD-ALS	59	M	0	0	0	N/A	N/A
**1**	P301L	52	M	3+	0	0	0	0
**2**	P301L	58	M	3+	0	0	0	0
**8**	P301L	58	M	3+	0	0	0	0
**6**	P301L	53	M	3+	0	0	0	0
**4**	P301L	72	M	3+	0	0	0	0
**5**	P301L	63	F	3+	0	0	0	0
**7**	P301L	61	F	3+	0	0	0	0
**9**	P301L	75	M	2+	0	0	0	0
**3**	P301L	56	M	3+	0	0	0	0
**12**	P301L	49	M	3+	0	N/A	0	0

GRN: progranulin mutation; P301L: P301L muation in MAPT
(a): Amyloid beta positive plaques in the outer and inner molecular layers, granular layer not affected.
(b): p-Tau positive neurites, isolated pretangle.
(c): argyrophilic grain disease
(d): positive p-Tau staining of the neuropil and moderate presence of pretangles and tangles.
Amyloid beta positive plaques throughout all layers of the dentate gyrus
(e): p-Tau positive neurites and pretangles; sparse amyloid beta positive plaques in the granular layer, and moderate in the outer and inner molecular layers, sparse cerebral amyloid angiopathy
(f): TDP43 positive cytoplasmic and sparse nuclear neuronal inclusions

Supplementary Table 3

Supplemental Tables: Summary statistical comparison of different CSA patterns of detergent-insoluble tau in TgTauP301L mice and in different phenotypes of FTLD. The P values were obtained with two-tailed generalized Wilcoxon test.

TgTauP301L Insol. tau

CSA Type	1	2	3	4
1	1			
2	<0.0001	1		
3	<0.0001	0.2423	1	
4	<0.0001	<0.0001	<0.0001	1

FTLD Insol. Tau

CSA Type	sv	bv*	bv
sv	1		
bv*	0.0055	1	
bv	0.0090	0.246	1

FTLD PK-resistant tau

CSA Type	sv	bv*	bv
sv	1		
bv*	0.9457	1	
bv	1.0000	0.9187	1

FTLD PK-sensistive tau

CSA Type	sv	bv*	bv
sv	1		
bv*	<0.0001	1	
bv	0.2722	0.17790	1
